# Pore Structure Regulation of RF-Derived Porous Carbons Using a Mixed-Level Design of Experiments for Lithium-Ion Battery Anode Materials

**DOI:** 10.3390/gels12090801

**Published:** 2026-09-02

**Authors:** Anrui Li, Yidan Tang, Shuo Yu, Shuli Yu, Le Sun, Qinsi Shao, Delun Zhu, Mengqian Wang, Ruicheng Bai

**Affiliations:** 1 School of Materials Science and Engineering, Shanghai University, Shanghai 200072, China; 2 State Key Laboratory of Advanced Refractories, Shanghai University, Shanghai 200444, China; 3 Shaoxing Institute of Technology, Shanghai University, Shaoxing 312000, China; 4 Institute for Sustainable Energy, School of Sciences, Shanghai University, Shanghai 200444, China; 5 Department of Polymer Science and Engineering, Zhejiang University, Hangzhou 310058, China; 6 Shanghai Institute of Applied Physics, Chinese Academy of Sciences, Shanghai 201800, China

**Keywords:** RF-derived porous carbons, Design of Experiments (DOE), pore-structure regulation, lithium-ion batteries, anode materials

## Abstract

Resorcinol–formaldehyde (RF)-derived porous carbons have attracted considerable attention as anode materials for lithium-ion batteries because of their tunable pore structures and continuous carbon frameworks. However, conventional one-factor-at-a-time experiments do not readily allow the relative effects of multiple preparation factors to be systematically compared within a unified experimental framework. In this study, a mixed-level Design of Experiments (DOE) was employed to systematically investigate the effects of solid content, gelation temperature, R/C ratio, combined gelation and acid-washing/aging times, and drying method on the BET specific surface area, total pore volume, and dominant pore size of RF-derived porous carbons. Representative preparation conditions were subsequently selected to prepare PC-1 and PC-2. Both samples exhibited predominantly amorphous mesoporous carbon structures and similar electrochemical response profiles. PC-1 exhibited a higher reversible specific capacity and slightly more favorable electrochemical kinetics. These concurrent observations suggest an association between the pore-structure characteristics and electrochemical behavior of the selected samples. PC-1 delivered an initial charge capacity of 416.67 mAh g^−1^ with an initial Coulombic efficiency of 79.31%. After 200 cycles at 0.1 A g^−1^, it retained a reversible capacity of 307.86 mAh g^−1^, corresponding to a capacity retention of 88.64% relative to the second-cycle charge capacity. These results indicate that, within the investigated design space, the DOE approach provides an exploratory basis for jointly comparing the statistical evidence and practical effect magnitudes of the preparation factors and for selecting representative candidates with favorable pore-structure characteristics. The integration of DOE-based factor screening with subsequent structural and electrochemical validation provides an experimentally grounded framework for relating preparation parameters to pore-structure responses and lithium-storage behavior, thereby supporting the rational development of RF-derived porous carbon anodes for lithium-ion batteries.

## 1. Introduction

Lithium-ion batteries (LIBs) have been widely used in portable electronic devices, electric vehicles, and large-scale energy-storage systems because of their high energy density, long cycle life, and well-established commercial foundation [1,2,3,4,5]. As the requirements for rate capability, cycling stability, and safety continue to increase, the development of anode materials with stable structures, rational ion-transport pathways, and tunable preparation processes remains of considerable importance [6,7,8,9]. Porous carbon materials have attracted extensive attention as anodes for LIBs owing to their good electronic conductivity, high structural stability, and tunable pore structures [10,11,12]. Among them, resorcinol–formaldehyde (RF)-derived porous carbons represent a typical class of three-dimensional porous carbon materials and are generally prepared through RF sol–gel polycondensation, drying, and subsequent high-temperature carbonization [13,14]. Their continuous carbon frameworks, tunable pore-size distributions, and good structural integrity can facilitate electrolyte wetting and Li^+^ transport while helping maintain the electrode structure, thereby showing promise as carbonaceous anode materials for lithium storage [15,16,17].

The pore structure of RF-derived porous carbons is highly sensitive to the preparation conditions [18]. The solid content affects the precursor concentration, sol–gel reaction process, and degree of network shrinkage, thereby altering the aggregation behavior of primary particles and the resulting pore architecture. The reaction temperature regulates the polycondensation rate and network growth. The molar ratio of resorcinol to catalyst (R/C) influences the catalytic environment, particle size, and framework connectivity. The gelation and acid-washing/aging durations determine the extent of further crosslinking within the three-dimensional network, impurity removal, and structural reinforcement. Meanwhile, the drying method directly affects the shrinkage, collapse, and preservation of the gel pore network during solvent removal. Collectively, these factors determine the specific surface area, pore volume, pore-size distribution, and framework morphology of the resulting porous carbons and consequently influence their lithium-storage behavior [19,20,21,22,23,24,25,26,27].

To date, most studies on RF-derived porous carbons have adopted a one-factor-at-a-time approach, in which individual preparation parameters, such as the R/C ratio, gelation temperature, drying conditions, or carbonization parameters, are varied separately to evaluate their effects on material structure or performance [28,29,30,31,32,33,34]. Although these studies have provided important insights into the role of individual synthesis variables, such an approach does not readily permit a direct quantitative comparison of the relative contributions of multiple preparation factors within a unified experimental framework. This limitation is particularly relevant to RF sol–gel systems, in which gel-network formation and subsequent pore development are influenced by multiple processing variables [35,36]. As a result, the dominant factors controlling specific pore-structure responses remain difficult to identify quantitatively, and the practical constraints associated with unfavorable combinations of preparation conditions are rarely considered systematically. RF-based carbon materials have also been investigated as anode materials for lithium-ion batteries, with previous studies mainly improving lithium-storage performance through regulation of individual material characteristics, including particle morphology, graphitization degree, pore structure, and heteroatom doping [37,38,39]. However, optimization of RF-derived porous carbons for lithium storage cannot be reduced to the maximization of a single structural parameter. An excessively high specific surface area may increase the electrode/electrolyte contact area, promote irreversible side reactions and solid electrolyte interphase formation, and consequently lower the initial Coulombic efficiency [40,41,42]. Similarly, excessively small or poorly interconnected pores may hinder electrolyte wetting and Li^+^ transport, whereas excessively large pores may reduce packing density and volumetric performance [43,44,45,46]. Despite these observations, previous studies have generally focused either on the effect of individual preparation variables or on the modification of specific material characteristics, while systematic studies that quantitatively rank multiple upstream processing factors, simultaneously balance several pore-structure responses, and subsequently evaluate the electrochemical performance of the selected candidates remain limited [47,48]. Therefore, establishing a more integrated relationship among preparation parameters, pore-structure responses, and lithium-storage performance through combined structural and electrochemical characterization is necessary for the rational process development of RF-derived porous carbon anodes [49].

**Figure 1:**
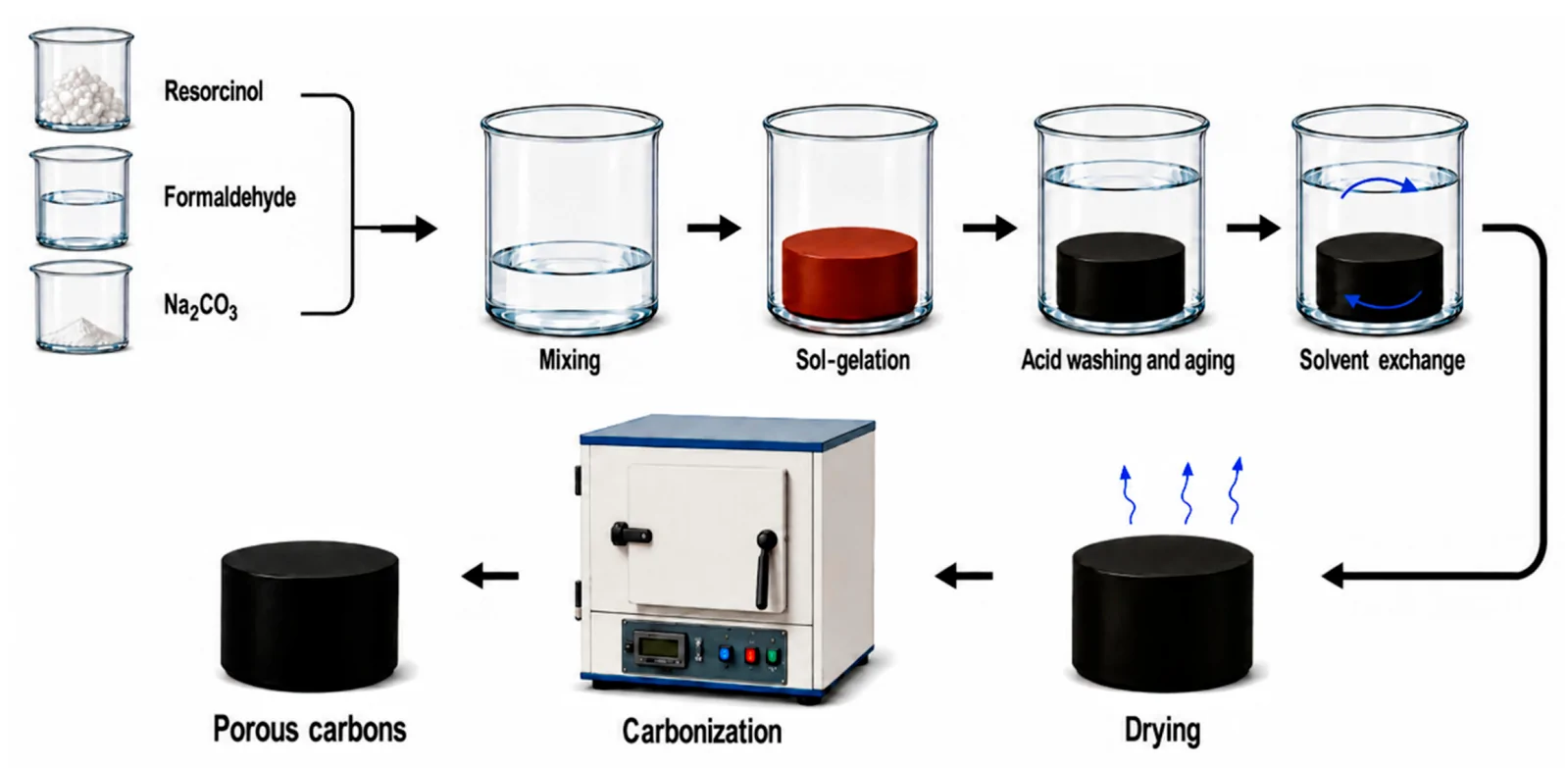
Schematic illustration of the preparation process for resorcinol–formaldehyde-derived porous carbons.

Accordingly, a mixed-level Design of Experiments (DOE) was employed to systematically investigate the effects of solid content, gelation temperature, R/C ratio, combined gelation and acid-washing/aging times, and drying method on the pore structure of RF-derived porous carbons. The overall experimental procedure is illustrated in Figure 1. Structural parameters, including the BET specific surface area, total pore volume, and pore-size distribution, were obtained from N_2_ adsorption–desorption measurements. Considering the pore-structure requirements of lithium-ion battery anodes, the governing trends and statistical significance of the investigated preparation factors were subsequently analyzed. Based on the DOE-assisted multi-response evaluation, two representative RF-derived porous carbons with relatively high BET specific surface areas, large total pore volumes, and appropriate mesoporous structures were selected for subsequent structural characterization and electrochemical evaluation. Both samples exhibited relatively high reversible specific capacities and stable cycling performance, demonstrating the lithium-storage viability of the selected porous-carbon candidates. SEM, TEM, XRD, Raman, and N_2_ sorption analyses further revealed similarities between the two samples in terms of particle morphology, carbon-framework structure, disordered-carbon characteristics, and mesopore distribution. Nevertheless, among the two selected candidates, the sample with a more open pore network and larger pore volume also exhibited a higher reversible capacity and slightly more favorable ion-transport behavior. Previous studies on RF-derived porous carbon anodes have generally sought to improve lithium-storage performance by regulating individual material characteristics, such as particle morphology, graphitization degree or heteroatom doping. In contrast, the mixed-level DOE employed in this work places greater emphasis on upstream process-factor screening rather than downstream material modification. The substantive advance of this approach lies in transforming multiple preparation variables that have conventionally been adjusted largely through empirical trial and error into a quantitatively ranked system of process factors. This enables the preparation parameters that predominantly govern key pore-structure responses to be identified while also revealing the practical constraints associated with gel formation under specific preparation conditions. Moreover, rather than using the maximization of a single structural parameter as the sole selection criterion, this study establishes a multi-response evaluation criterion tailored to the requirements of lithium-ion battery anodes, comprehensively balancing the BET specific surface area, total pore volume, pore size, and processing practicality. By further integrating process screening with sample re-preparation, structural characterization, and electrochemical performance evaluation, the DOE analysis is connected with the application performance of the selected representative candidates. Therefore, the principal contribution of this work is the establishment of an integrated screening framework linking preparation parameters, pore-structure responses, representative-candidate selection, and lithium-storage performance evaluation. This framework enables the relative importance of upstream preparation variables to be identified and provides a systematic approach for experimental process screening and application-oriented candidate selection of RF-derived porous carbons.

## 2. Results and Discussion

### 2.1. DOE-Based Analysis of Pore Structure Regulation in RF-Derived Porous Carbons

A mixed-level DOE was employed to simultaneously investigate the effects of nominal solid content, gelation temperature, R/C ratio, combined gelation and acid-washing/aging times, and drying method on the pore structure of RF-derived porous carbons. The analysis first evaluated the feasibility and repeatability of sample preparation. Subsequently, a main-effects-only linear model was used to compare the relative influence of each factor on the BET specific surface area, total pore volume, and dominant pore size. The DOE was designed primarily to identify and rank the dominant preparation factors. Accordingly, the main-effects model was used to characterize the average effects of the investigated factors within the experimental design space, while possible interaction effects were considered a limitation of the present analysis and were not incorporated into the interpretation of individual main effects. Representative candidate conditions were then selected using a multi-response scoring scheme tailored to the pore-structure requirements of lithium-ion battery anodes.

Of the 36 experiments performed, 31 successfully formed gels and yielded valid pore-structure data, whereas five failed to form gels. To distinguish process feasibility from the continuous material-property responses, gel formation was treated as a separate binary response, with successful gelation coded as 1 and gelation failure coded as 0. The factor-level distributions of successful and failed runs and the corresponding gelation success rates are summarized in Table S1. The overall gelation success rate was 86.1%. The factor-wise exact analysis showed that deionized water volume exhibited the clearest association with gelation outcome within the investigated design space (Fisher–Freeman–Halton exact *p* = 0.0080). The gelation success rate decreased from 100.0% at 9 mL of water to 90.9% at 20 mL and 60.0% at 40 mL. The R/C ratio also exhibited an apparent factor-level association with gelation outcome (exploratory exact *p* = 0.0499), with success rates of 100.0%, 100.0%, 77.8%, and 62.5% at R/C ratios of 100, 200, 400, and 1000, respectively. However, all three failures observed at R/C = 1000 occurred simultaneously at a water volume of 40 mL. Moreover, all three experimental runs combining 40 mL of water with R/C = 1000 failed to form gels. Therefore, the apparent R/C-related trend cannot be interpreted independently of the low-solid-content condition and may reflect a combination-dependent feasibility constraint. No similarly clear factor-level associations were observed for drying method, gelation temperature, or the combined gelation and acid-washing/aging times. The five absent pore-structure datasets were therefore not considered to be missing completely at random but instead resulted from process failure in specific regions of the experimental design space. Because the BET specific surface area, total pore volume, and dominant pore size are structurally undefined when a gel does not form, these responses were not statistically imputed. The continuous-response models were fitted only to successfully gelled samples. Consequently, the estimated main effects on the continuous pore-structure responses are conditional on successful gelation and may be affected by factor-dependent selection bias, particularly in the low-solid-content and high-R/C region. These effects should therefore not be interpreted as unconditional estimates across the entire design space. Instead, gelation feasibility was considered separately alongside the continuous pore-structure responses when evaluating the preparation conditions.

The replicate results presented in Table S2 show that, for the ambient-pressure-dried replicate pair (Runs 33 and 34), the coefficients of variation for the BET specific surface area, total pore volume, and dominant pore size were 4.06%, 0.69%, and 0.52%, respectively. For the freeze-dried replicate pair (Runs 35 and 36), the corresponding coefficients of variation were 2.41%, 6.24%, and 2.11%, respectively. These results indicate relatively small variations in the BET specific surface area and dominant pore size under the selected replicate conditions. Although the total pore volume of the freeze-dried samples exhibited comparatively greater variability, it remained acceptable for preliminary screening. Because only one set of replicate conditions was included for each drying method, these replicate results primarily reflect the experimental error in the vicinity of the corresponding local process conditions.

The adequacy of the main-effects models was further evaluated before interpreting the individual factor effects. As summarized in Table S3, the models for the log-transformed BET specific surface area and total pore volume yielded R^2^ values of 0.701 and 0.746 and adjusted R^2^ values of 0.528 and 0.599, respectively, indicating moderate explanatory capability. Their predicted R^2^ values were lower, at 0.202 and 0.302, suggesting limited predictive capability for untested factor combinations. The pore-size model showed a lower adjusted R^2^ of 0.388 and a negative predicted R^2^ (−0.127), indicating that it should be interpreted primarily as an exploratory model. Residual diagnostics showed no marked deviations from normality or homoscedasticity for the BET surface-area and total-pore-volume models, whereas the pore-size model was more strongly affected by the unusually high value of Run 11. Significant lack-of-fit was observed for all three models; however, because the pure-error estimate was based on only two degrees of freedom, the lack-of-fit results were interpreted cautiously. Accordingly, the DOE models were used primarily for main-effect screening and ranking of experimentally investigated conditions rather than for precise prediction of a global optimum.

The analysis of variance (ANOVA) results are summarized in Table S4. The R/C ratio was the dominant factor affecting the BET specific surface area and total pore volume, with contribution rates of 39.5% and 39.7% and corresponding *p*-values of 0.0013 and 0.0006, respectively. Gelation temperature contributed 25.2% and 28.6% to these two responses, respectively, and was also statistically significant. These results suggest that the catalyst proportion and gelation temperature play major roles in regulating the polycondensation, crosslinking, and framework growth of the RF precursor, thereby influencing the extent of pore development after carbonization. In comparison, water volume/nominal solid content, the combined gelation and acid-washing/aging times, and drying method exerted relatively weaker effects on the BET specific surface area and total pore volume. For the dominant pore size, the combined gelation and acid-washing/aging times were the only statistically significant factors, with a contribution rate of 24.0% and a *p*-value of 0.0365. The R/C ratio showed marginal statistical significance, with a contribution rate of 19.0% and a *p*-value of 0.0709. Overall, the R/C ratio and gelation temperature were the dominant factors governing the BET specific surface area and total pore volume. For the dominant pore size, the combined gelation and acid-washing/aging times showed a statistically significant main effect in the present dataset; however, because the pore-size model exhibited relatively weak predictive performance and was sensitive to the unusually high value of Run 11, this effect should be interpreted as a dataset-specific trend rather than a robust predictive relationship. The contribution rates reported here therefore represent the relative contributions of the main effects within the adopted main-effects model and should not be interpreted as accounting for all sources of systematic variation in the pore-structure responses. Possible two-factor interactions may contribute to response variations that are not captured by the present main-effects model, particularly for experimental conditions showing deviations from the factor-level mean trends. Accordingly, the individual factor effects identified here are interpreted primarily as average screening effects within the investigated design space rather than as independent mechanistic effects or complete descriptions of the combined processing-variable relationships. Statistical significance and practical significance were distinguished when interpreting these factor rankings. For the BET specific surface area and total pore volume, the R/C ratio and gelation temperature exhibited both statistically significant effects and relatively large contribution rates, indicating that they were practically influential factors within the investigated design space. However, the increased risk of unsuccessful gelation at R/C = 1000 demonstrates that a favorable main-effect trend does not necessarily correspond to a practically optimal condition. For the dominant pore size, the combined gelation and acid-washing/aging time showed a statistically significant main effect; nevertheless, because of the weak predictive performance of the pore-size model and its sensitivity to Run 11, this result is interpreted as a dataset-specific screening trend. The R/C ratio showed only marginal statistical significance for dominant pore size despite its contribution rate of 19.0% and was therefore not classified as statistically significant. Conversely, nominal solid content and drying method remained practically relevant because of their respective influences on gelation feasibility and processing accessibility, even though they did not exhibit strong statistical effects on the continuous pore-structure responses. Accordingly, the final factor interpretation and candidate selection jointly considered statistical evidence, observed response magnitudes, gelation feasibility, and processing practicality rather than relying on *p*-values or contribution rates alone.

The response analysis of the BET specific surface area identified the R/C ratio and gelation temperature as the principal influencing factors. As shown in Table S5, increasing the R/C ratio from 100 to 1000 increased the mean BET specific surface area from 91.6 to 575.1 m^2^ g^−1^. This result suggests that decreasing the catalyst content may promote pore development by slowing the polycondensation rate and altering the growth and packing behavior of the primary particles. However, the risk of unsuccessful gelation was relatively high at R/C = 1000; therefore, pore-structure development must be balanced against gelation stability. Increasing the gelation temperature from 40 to 80 °C increased the mean BET specific surface area from 132.9 to 386.2 m^2^ g^−1^, indicating that a higher temperature favors the formation of the RF network. The effect of the combined gelation and acid-washing/aging times was non-monotonic, with the mean values following the order of (16 + 8) h > (48 + 24) h > (24 + 12) h > (8 + 4) h. Overall, a higher R/C ratio, a higher gelation temperature, and an intermediate time combination were more favorable for increasing the BET specific surface area, whereas nominal solid content and drying method played comparatively secondary roles.

The response trend of the total pore volume was generally consistent with that of the BET specific surface area. As shown in Table S6, the R/C ratio and gelation temperature remained the principal influencing factors. The highest mean total pore volumes were obtained at R/C = 1000 and a gelation temperature of 80 °C, reaching 0.6474 and 0.4743 cm^3^ g^−1^, respectively, in agreement with the ANOVA results. However, the factor level associated with the highest mean response does not necessarily correspond to the individual sample with the largest measured pore volume. In this study, Run 24 exhibited the highest measured total pore volume of 1.475 cm^3^ g^−1^ under conditions of 34.3 wt.% nominal solid content, 80 °C, R/C = 400, and a combined gelation and acid-washing/aging time of (48 + 24) h. This result suggests that, while the main-effect analysis successfully captures the overall response trends of the principal factors, specific combinations of preparation conditions may provide additional contributions to pore development. Such combination-dependent behavior may also reflect possible interaction-related effects and should therefore be considered when interpreting individual experimental runs. Accordingly, the present DOE analysis uses the main-effect trends primarily for factor screening and ranking, while the actual measured responses of individual runs are also considered in the subsequent selection of representative candidates.

The response analysis of the dominant pore size is presented in Table S7. The factor-level means indicate that the combined gelation and acid-washing/aging time of (24 + 12) h, R/C = 1000, a nominal solid content of 22.1 wt.%, and a gelation temperature of 40 °C were associated with relatively large mean dominant pore sizes. However, the dominant pore size of Run 11 was substantially larger than those of the other samples, disproportionately increasing the mean responses at the corresponding factor levels. Therefore, the pore-size trend cannot be determined solely from the mean responses. Overall, the dominant pore sizes of most samples were concentrated within 3–5 nm, whereas only a few samples exhibited values within 9–14 nm, indicating that the RF-derived porous carbons were dominated by relatively small mesopores. Appropriately sized mesopores can facilitate electrolyte wetting and Li^+^ transport, whereas excessively large pores may reduce the packing density and volumetric performance of the material. Therefore, representative samples should be selected by jointly considering the BET specific surface area, total pore volume, and dominant pore size rather than by pursuing a larger pore size alone. Samples combining a relatively high specific surface area, large pore volume, and appropriate mesopore distribution are more suitable for subsequent structural characterization and electrochemical evaluation.

To avoid selecting samples solely on the basis of high specific surface area and pore volume, the DOE samples were evaluated using a multi-response desirability function tailored to the requirements of lithium-ion battery anodes. The screening criteria used in the multi-response evaluation were selected by considering the pore-structure ranges commonly associated with favorable lithium-storage behavior in porous-carbon anodes, together with the need to balance the different functional roles of specific surface area, pore volume, and pore size [50,51,52,53]. A relatively high BET specific surface area and sufficient pore volume were considered beneficial for increasing the accessible electrode/electrolyte interface and facilitating electrolyte infiltration, whereas an appropriate mesoporous pore-size range was preferred to promote Li^+^ transport without excessively compromising electrode-packing characteristics. Based on these considerations, 700 m^2^ g^−1^, 0.6 cm^3^ g^−1^, and 3–10 nm were adopted as practical reference criteria for the BET specific surface area, total pore volume, and dominant pore size, respectively. These values were used for application-oriented multi-response screening rather than being regarded as universally optimal pore-structure parameters. As shown in Table 1, Run 24 achieved the highest overall desirability score and was therefore selected as the primary candidate based on the multi-response pore-structure evaluation. This sample exhibited a BET specific surface area of 737.5949 m^2^ g^−1^, a total pore volume of 1.4750 cm^3^ g^−1^, and a dominant pore size of 9.2353 nm, indicating a favorable overall balance among the evaluated pore-structure characteristics. In contrast, Run 7 was not selected solely according to its overall desirability ranking. Rather, it was selected as a secondary, application-oriented candidate by additionally considering its relatively favorable pore-structure characteristics and the practical advantage of ambient-pressure drying. Thus, the selection of Run 24 primarily reflects the multi-response desirability evaluation, whereas Run 7 represents an alternative preparation route selected by additionally incorporating processing practicality. Because a separate sensitivity analysis using alternative target or weighting settings was not performed, the desirability ranking is interpreted specifically within the predefined screening criteria and equal-weighting scheme used in the present study, rather than being regarded as invariant to alternative desirability specifications.

### 2.2. Structural Characterization of Selected RF-Derived Porous Carbons

Based on the multi-response evaluation and the additional consideration of processing practicality, Run 24 was selected as the primary candidate because it achieved the highest overall desirability score. Run 7 was selected as an alternative candidate by jointly considering its relatively favorable pore-structure characteristics and the practical advantage of ambient-pressure drying. Because ambient-pressure drying requires less specialized equipment than freeze drying, Run 7 provides a practically relevant alternative preparation route for comparison with the freeze-dried Run 24. The detailed preparation conditions for Runs 24 and 7 are summarized in Table 2.

**Figure 2:**
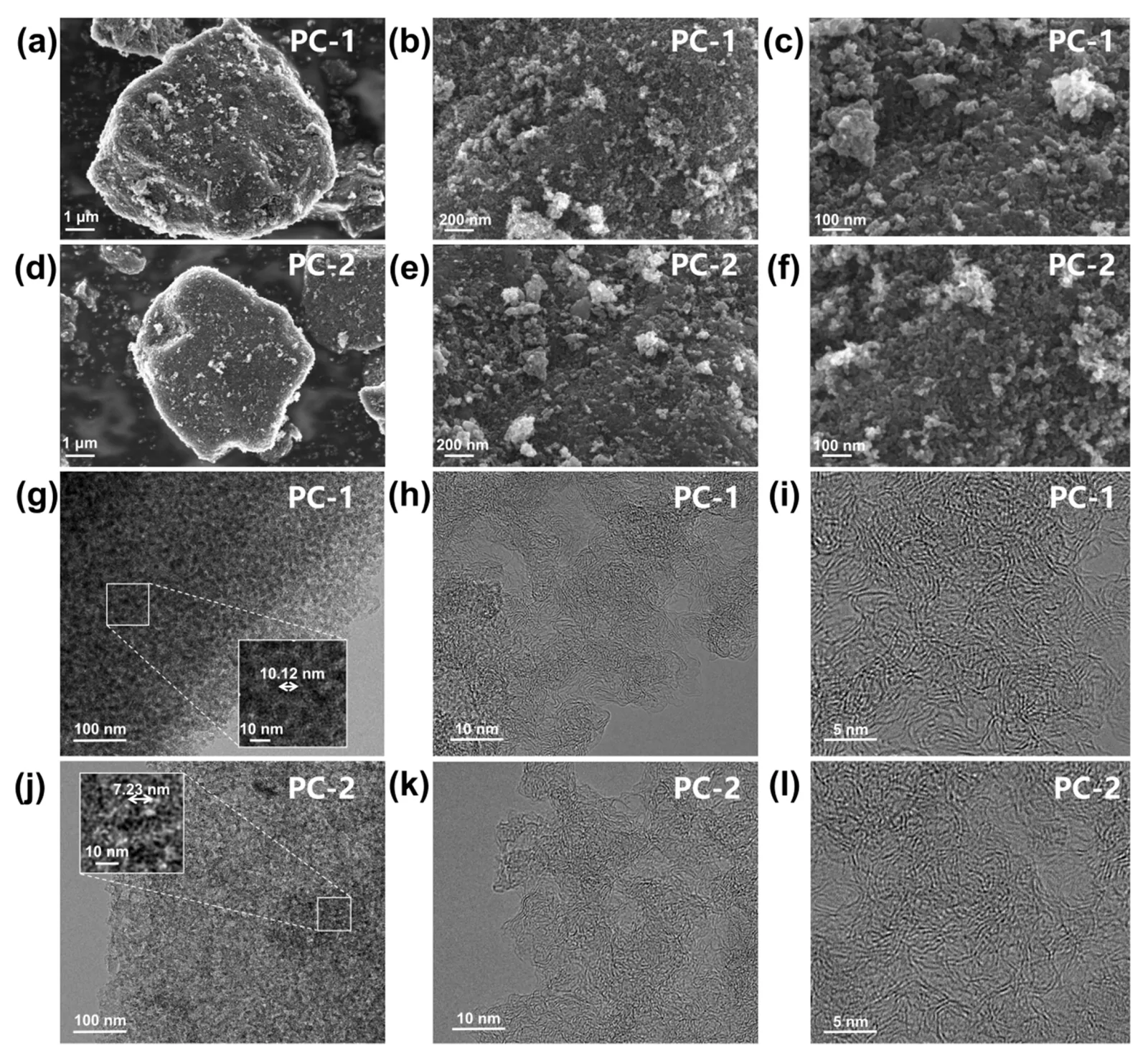
Morphological and microstructural characterization of PC-1 and PC-2. (**a**–**c**) SEM images of PC-1 with scale bars of 1 μm, 200 nm, and 100 nm, respectively; (**d**–**f**) SEM images of PC-2 with scale bars of 1 μm, 200 nm, and 100 nm, respectively; (**g**) TEM image of PC-1 with a scale bar of 100 nm; (**h**,**i**) HRTEM images of PC-1 with scale bars of 10 nm and 5 nm, respectively; (**j**) TEM image of PC-2 with a scale bar of 100 nm; (**k**,**l**) HRTEM images of PC-2 with scale bars of 10 nm and 5 nm, respectively.

Scanning electron microscopy (SEM) was used to examine the particle morphology and surface characteristics of PC-1 and PC-2. As shown in Figure 2a,d, both samples exhibit irregular particulate morphologies and retain relatively intact particle structures after carbonization. Based on an approximate estimation from the SEM scale bars, the representative particle sizes of PC-1 and PC-2 are approximately 8.0 and 6.9 μm, respectively, indicating that both samples consist predominantly of micrometer-sized carbon particles. Compared with PC-2, PC-1 displays a rougher surface with more visible depressions, fissures, and open pores, whereas PC-2 exhibits a comparatively smoother surface. At higher magnifications (Figure 2b,c,e,f), both samples consist of interconnected nanoscale carbon building blocks forming continuous porous frameworks. PC-1 shows greater exposed porosity and more distinct interconnected pore features than PC-2, consistent with its more open porous structure.

Transmission electron microscopy (TEM) was employed to further examine the nanoscale pore structure within the carbon frameworks and the local structural differences between the two samples. As shown in Figure 2g,j, both samples consist of interconnected nanoscale carbon building blocks that form continuous porous frameworks. Irregular pore boundaries and pronounced contrast variations are observed near the framework edges. Compared with PC-2, PC-1 exhibits rougher carbon-framework edges and stronger local contrast variations, reflecting more complex pore boundaries and greater local structural heterogeneity. To further compare their local pore structures, three representative pores with relatively well-defined boundaries were selected from the TEM images of each sample. The measured pore diameters of PC-1 were 10.12, 10.59, and 10.32 nm, giving a mean diameter of approximately 10.34 nm. The corresponding values for PC-2 were 7.23, 8.11, and 8.24 nm, with a mean diameter of approximately 7.86 nm. These locally measured pore dimensions were within a similar size range to the pore-size values obtained from the subsequent N_2_ sorption analysis. High-resolution transmission electron microscopy (HRTEM) was further used to investigate the arrangement of the carbon layers and the degree of local structural ordering. As shown in Figure 2h,k and Figure 2i,l, both PC-1 and PC-2 are composed predominantly of disordered carbon networks, and no continuous long-range-ordered lattice structure is observed, indicating that both RF-derived porous carbons are primarily amorphous. Nevertheless, curved and discontinuous carbon fringes can be identified in some local regions, suggesting the presence of short-range-ordered, graphitic-like nanodomains. Based on the relatively distinguishable carbon fringes in the HRTEM images, the local interlayer spacings of PC-1 and PC-2 were estimated to be approximately 0.39 and 0.37 nm, respectively. The slightly larger local interlayer spacing of PC-1 suggests a relatively looser stacking arrangement of the carbon layers. Compared with PC-2, PC-1 exhibits more clearly defined local carbon fringes, indicating more pronounced local short-range ordering while retaining an overall amorphous carbon framework.

Taken together, the SEM and TEM results show that both PC-1 and PC-2 consist of interconnected nanoscale carbon building blocks that form continuous particulate porous carbon frameworks, with highly similar overall morphologies and microstructures. In comparison, PC-1 exhibits more pronounced surface undulations, open pore features, hierarchical framework characteristics, and locally short-range-ordered carbon fringes.

**Figure 3:**
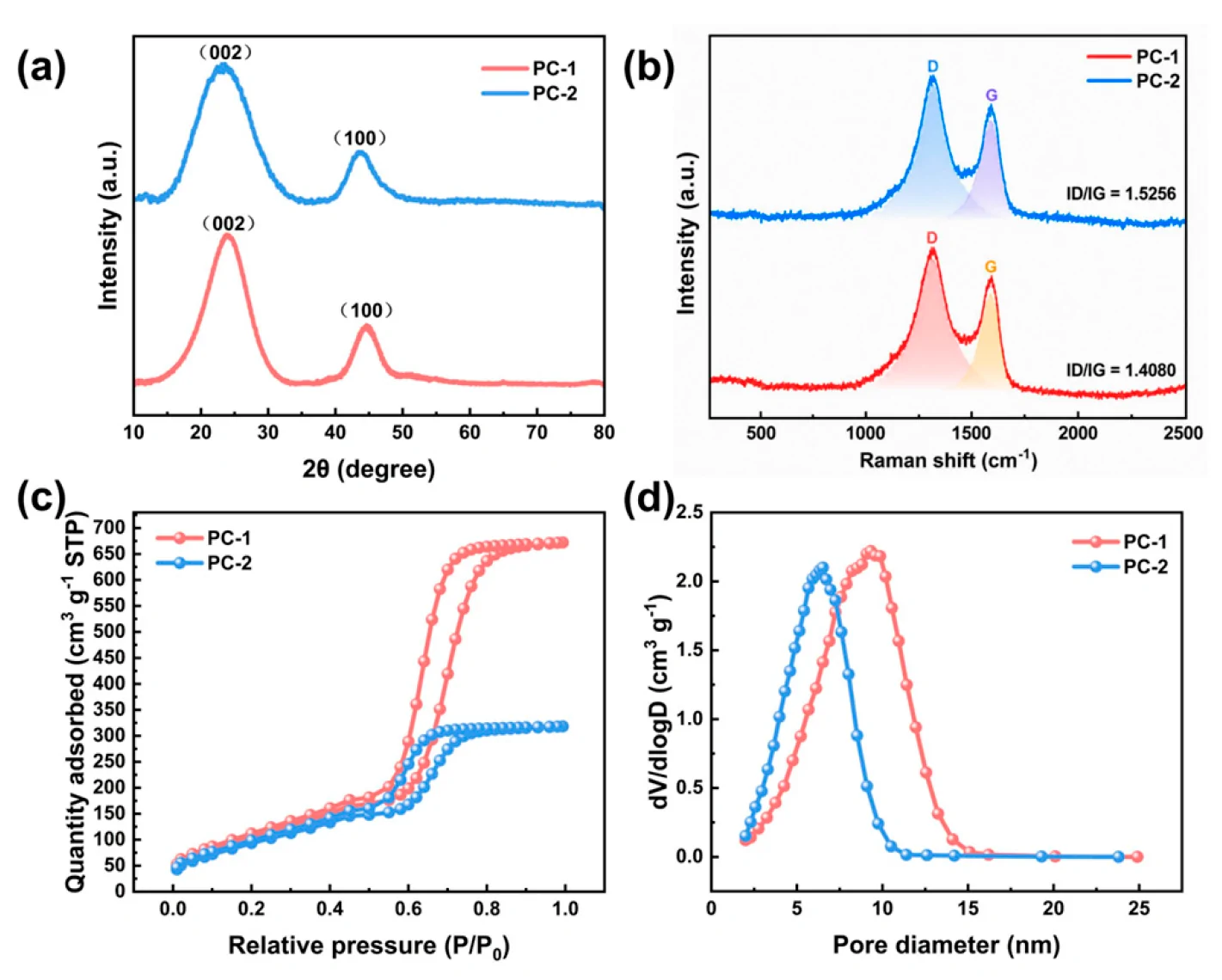
Carbon-structure and pore-structure characterization of PC-1 and PC-2. (**a**) XRD patterns; (**b**) Raman spectra; (**c**) N_2_ adsorption–desorption isotherms; and (**d**) Barrett–Joyner–Halenda (BJH) pore-size distributions of PC-1 and PC-2.

X-ray diffraction (XRD) was performed to further investigate the carbon structure and degree of structural ordering of PC-1 and PC-2. As shown in Figure 3a, both samples exhibit broad diffraction features at approximately 23° and 43–45°, which can be assigned to the (002) and (100) reflections of turbostratic carbon, respectively. No sharp crystalline diffraction peaks are observed, indicating that both samples are dominated by amorphous carbon with a low degree of graphitization. The (002) peaks of PC-1 and PC-2 are located at 23.13° and 23.80°, corresponding to d_(002)_ values of 0.3842 and 0.3736 nm, respectively. Both values are larger than the interlayer spacing of ideal graphite (0.3354 nm), suggesting relatively loose stacking of the carbon layers. Compared with PC-2, PC-1 exhibits a larger d_(002)_ value, indicating a greater average separation and a less compact arrangement of adjacent carbon layers. This difference in d_(002)_ primarily describes the average interlayer configuration rather than the density of defects within the carbon framework. In addition, the (002) diffraction peak of PC-2 appears relatively broader than that of PC-1, which may be associated with differences in their local carbon-layer arrangements. Combined with the broad (002) and (100) diffraction features, the XRD results indicate that both samples are predominantly amorphous carbon materials containing local short-range-ordered domains.

Raman spectroscopy was performed to evaluate the defect density and structural disorder of PC-1 and PC-2. As shown in Figure 3b, both samples exhibit characteristic D and G bands at approximately 1350 and 1590 cm^−1^, respectively. The D band is associated with defect sites in disordered carbon structures, whereas the G band corresponds to the in-plane vibration of sp^2^-hybridized carbon atoms. The calculated ID/IG ratios of PC-1 and PC-2 are 1.4080 and 1.5256, respectively. Under the same measurement and fitting conditions, a higher ID/IG ratio generally indicates a stronger contribution from defect-activated scattering and a higher density of Raman-active defects within the local sp^2^ carbon network. Accordingly, the Raman results suggest that PC-2 contains more defect-related structural features than PC-1. Both RF-derived porous carbons show abundant defect features and low degrees of graphitization, consistent with the structural characteristics of typical amorphous carbon materials. The XRD, Raman, TEM, and HRTEM observations therefore provide complementary information on the interlayer arrangement, local defect structure, and short-range carbon-layer characteristics of the two RF-derived porous carbons.

N_2_ adsorption–desorption measurements were conducted to quantitatively characterize the accessible pore structures of PC-1 and PC-2, as shown in Figure 3c,d. Both samples exhibit relatively limited adsorption in the low-relative-pressure region, indicating a comparatively small micropore contribution. The pronounced hysteresis loops in the medium- to high-relative-pressure region demonstrate that mesopores constitute the dominant accessible pore system in both samples. The dominant pore size of PC-1 and PC-2 are 10.4436 and 8.0128 nm, respectively. Additional NLDFT analysis also confirms that the pore-size distributions of both samples are mainly concentrated in the mesoporous range, with PC-1 exhibiting a relatively larger characteristic pore size than PC-2 (Figure S1). PC-1 exhibits a BET specific surface area of 693.8131 m^2^ g^−1^ and a total pore volume of 1.2982 cm^3^ g^−1^, compared with 613.1626 m^2^ g^−1^ and 0.4924 cm^3^ g^−1^ for PC-2. These quantitative results demonstrate that PC-1 provides a larger N_2_-accessible surface area, a substantially greater pore volume, and a larger average mesopore size, while both samples remain predominantly mesoporous.

**Figure 4:**
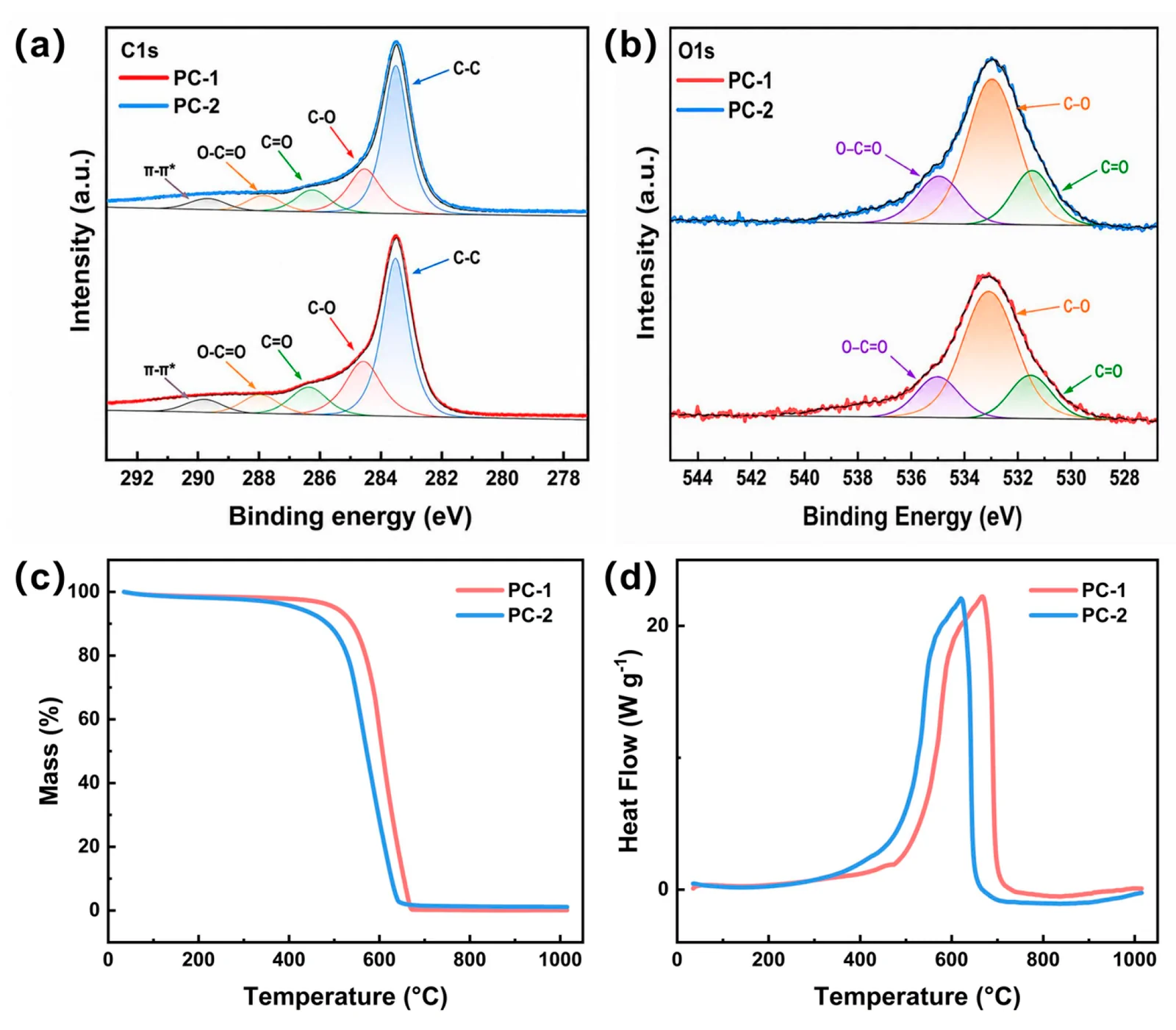
High-resolution XPS spectra and thermal behavior of PC-1 and PC-2: (**a**) deconvoluted C1s spectra, (**b**) deconvoluted O1s spectra, (**c**) TG curves, and (**d**) DSC curves under an air atmosphere.

X-ray photoelectron spectroscopy (XPS) was employed to characterize the surface chemical states of PC-1 and PC-2. Both samples are predominantly composed of C and O, with C and O contents of 91.39 and 8.61 at.% for PC-1 and 91.91 and 8.09 at.% for PC-2, respectively, indicating comparable surface elemental compositions. As shown in Figure 4a, the high-resolution C 1s spectra can be deconvoluted into components centered at approximately 284.8, 286.0, 287.5, 288.9, and 290–291 eV, assigned to C–C/C=C, C–O, C=O, O–C=O, and π–π*, respectively, with C–C/C=C being the dominant contribution. The corresponding O 1s spectra in Figure 4b can be resolved into three components at approximately 531.9, 533.2–533.3, and 534.9 eV, corresponding to C=O, C–O, and O–C=O/high-binding-energy oxygen species, respectively. Based on the relative areas of the fitted components, the C–C/C=C, C–O, C=O, O–C=O, and π–π components accounted for approximately 87.6%, 4.5%, 2.4%, 1.0%, and 4.5% of the C 1s envelope for PC-1 and 87.2%, 3.7%, 2.7%, 1.0%, and 5.4% for PC-2, respectively. For the O 1s spectra, the C=O, C–O, and O–C=O components accounted for approximately 27.0%, 50.6%, and 22.4% for PC-1 and 32.1%, 44.0%, and 23.9% for PC-2, respectively. C–C/C=C was the predominant carbon component, whereas C–O was the major oxygen-containing component in both samples. The comparable C 1s and O 1s component distributions suggest broadly similar surface chemical environments for PC-1 and PC-2. Therefore, their electrochemical differences may be associated with combined variations in pore structure, carbon-framework characteristics, and ion-transport behavior.

As shown in Figure 4c,d, the thermal behavior of PC-1 and PC-2 was evaluated by TG–DSC under an air atmosphere. Both samples exhibited only slight mass loss at relatively low temperatures, retaining approximately 98.28% and 97.61% of their initial masses at 300 °C for PC-1 and PC-2, respectively. With increasing temperature, pronounced oxidative mass loss occurred as a result of combustion of the carbon framework. The temperature corresponding to 5% mass loss (T5) was approximately 503 °C for PC-1, notably higher than that of PC-2 (418 °C), while both samples approached nearly complete mass loss at higher temperatures. The DSC curves showed corresponding high-temperature exothermic behavior, with the main exothermic peaks located at approximately 666.4 °C for PC-1 and 620.3 °C for PC-2. The higher T5 value and the positive shift in the major exothermic peak for PC-1 are consistent with each other, indicating that the principal oxidation process of PC-1 occurs at higher temperatures. Overall, these results demonstrate that PC-1 possesses relatively greater oxidative thermal stability than PC-2.

To further evaluate batch-to-batch reproducibility, PC-1 and PC-2 were independently re-prepared three times under the conditions corresponding to DOE-selected Runs 24 and 7, respectively. The re-prepared PC-1 samples exhibited a BET specific surface area of 693.8131 ± 12.7209 m^2^ g^−1^, a total pore volume of 1.2982 ± 0.1081 cm^3^ g^−1^, and a dominant pore size of 10.4436 ± 0.2204 nm (mean ± standard deviation, *n* = 3). The re-prepared PC-2 samples exhibited a BET specific surface area of 613.1626 ± 24.8944 m^2^ g^−1^, a total pore volume of 0.4924 ± 0.0303 cm^3^ g^−1^, and a dominant pore size of 8.0128 ± 0.0417 nm (*n* = 3). The BET specific surface area and total pore volume therefore exhibited relatively limited batch-to-batch variations, whereas the dominant pore size, particularly that of PC-2, showed greater sensitivity to sample re-preparation. Nevertheless, all independently prepared samples remained predominantly mesoporous, and the relative pore-structure relationship between PC-1 and PC-2 was maintained. These quantitative results provide a more direct assessment of the reproducibility and remaining batch-to-batch variability of the selected synthesis conditions.

### 2.3. Lithium-Storage Performance of RF-Derived Porous Carbons

**Figure 5:**
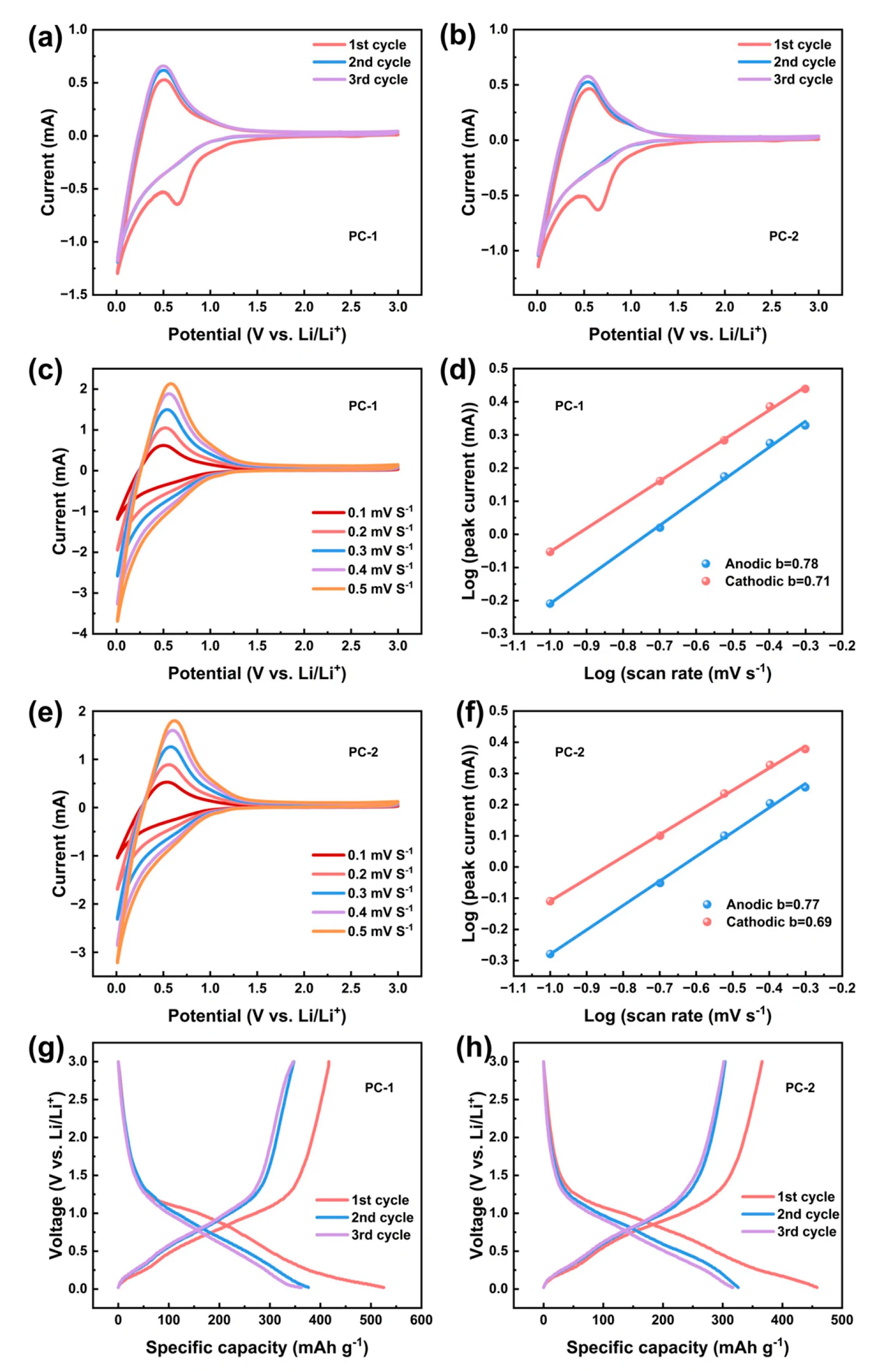
Initial electrochemical behavior and kinetic analysis of PC-1 and PC-2 in lithium-ion half-cells. (**a**,**b**) CV curves of PC-1 and PC-2, respectively, during the first three cycles at 0.1 mV s^−1^; (**c**,**e**) CV curves of PC-1 and PC-2, respectively, at different scan rates from 0.1 to 0.5 mV s^−1^; (**d**,**f**) corresponding log(i)–log(v) plots for determining the b values of PC-1 and PC-2, respectively; and (**g**,**h**) galvanostatic charge–discharge profiles of PC-1 and PC-2, respectively, during the first three cycles.

To evaluate the lithium-storage performance of the representative candidates selected through the DOE-based screening strategy, PC-1 and PC-2 were investigated as anode materials for lithium-ion batteries. Figure 5a,b show the CV curves of PC-1 and PC-2, respectively, during the first three cycles at a scan rate of 0.1 mV s^−1^. The two samples exhibit similar curve profiles, suggesting broadly similar lithium-storage mechanisms. The irreversible reduction peak during the first cathodic scan is mainly associated with electrolyte decomposition, SEI formation, and irreversible Li^+^ reactions with high-energy defect sites. The broad redox response in the low-potential region indicates that lithium storage involves Li^+^ adsorption/desorption at defects, edges, and pore-wall sites, together with insertion/extraction between locally short-range-ordered carbon layers. The near-overlap of the second and third cycles suggests that the SEI becomes relatively stable after the initial interfacial reactions and that the subsequent lithium-storage processes are highly reversible. Although PC-2 exhibits a higher degree of structural disorder, PC-1 shows a stronger current response under the same testing conditions. This stronger current response is consistent with the more open mesoporous network and larger pore volume of PC-1 and may reflect the combined contributions of pore accessibility and other physicochemical characteristics to electrolyte wetting and Li^+^ transport [54]. To further investigate the lithium-storage kinetics of PC-1 and PC-2, CV measurements were performed at scan rates of 0.1, 0.2, 0.3, 0.4, and 0.5 mV s^−1^, as shown in Figure 5c,e. As the scan rate increased, the current responses of both electrodes gradually intensified, accompanied by an expansion of the enclosed CV area. Meanwhile, the curves retained similar overall profiles at different scan rates, indicating that both electrodes maintained relatively stable lithium-storage reactions within the investigated scan-rate range. The oxidation peaks shifted toward more positive potentials with increasing scan rate, reflecting the gradual increase in electrode polarization. To evaluate the kinetic characteristics, the relationship between the peak current (*i*) and scan rate (*v*) was analyzed using Equations (1) and (2), where a and b are adjustable parameters.

(1) $$i=av^{b}$$

(2) logi=blogv+loga

The b value was obtained from the slope of the linear fitting of log(i) versus log(v), as shown in Figure 5d,f. Generally, a b value close to 0.5 indicates a diffusion-controlled process, whereas a value close to 1.0 represents a surface-controlled capacitive process. The fitted b values for the anodic and cathodic peaks of PC-1 were 0.78 and 0.71, respectively, while the corresponding values for PC-2 were 0.77 and 0.69. All the b values lie between 0.5 and 1.0, demonstrating that the lithium-storage kinetics of both electrodes are jointly governed by diffusion-controlled Li^+^ insertion/extraction and surface-controlled capacitive processes [55]. The slightly higher b values of PC-1 suggest a marginally greater capacitive contribution and a somewhat more favorable kinetic response, whereas the small differences between the two samples indicate that their overall lithium-storage mechanisms are similar [56].

Following the CV analysis, galvanostatic charge–discharge measurements were conducted to quantitatively evaluate the initial capacities and electrochemical reversibility of the two samples. As shown in Figure 5g,h, the lower cutoff voltage was set to 0.01 V to access the lithium-storage sites associated with defects, pore walls, and short-range ordered carbon layers in the low-potential region, thereby enabling a more comprehensive evaluation of the reversible lithium-storage capacity. Meanwhile, this cutoff avoided the possible metallic lithium deposition and intensified interfacial side reactions associated with further decreases in potential. PC-1 delivered first-cycle discharge and charge capacities of 525.37 and 416.67 mAh g^−1^, respectively, corresponding to an initial Coulombic efficiency (ICE) of 79.31%. The corresponding values for PC-2 were 458.01 and 365.94 mAh g^−1^, with an ICE of 79.90%. The first-cycle charge capacities and ICE values obtained from three independently assembled cells were 416.6 ± 6.32 mAh g^−1^ and 79.3 ± 0.38% for PC-1, and 365.9 ± 5.7 mAh g^−1^ and 79.9 ± 0.41% for PC-2, respectively (*n* = 3). During the initial discharge, reversible lithium-storage reactions were accompanied by electrolyte reduction and decomposition, SEI film formation, and the irreversible consumption of Li^+^. This portion of Li^+^ could not be completely extracted during the subsequent charging process, resulting in an initial discharge capacity substantially higher than the corresponding charge capacity. Although PC-1 possesses a larger specific surface area and a higher absolute irreversible capacity, its ICE remains close to that of PC-2. Both samples exhibit continuously sloping voltage profiles, consistent with the broad redox responses observed in the CV curves. This behavior further suggests that Li^+^ storage is not governed by a single type of site but may involve adsorption at defects, edges, and pore-wall surfaces, accompanied by insertion/extraction between locally short-range-ordered carbon layers [57]. In the second cycle, PC-1 delivered discharge and charge capacities of 376.18 and 347.32 mAh g^−1^, respectively, higher than the corresponding values of 326.00 and 304.69 mAh g^−1^ for PC-2. The gradual overlap of the second- and third-cycle profiles is consistent with the CV results, further confirming electrochemical reversibility for both electrodes following the initial cycle and stable lithium-storage behavior during subsequent cycling.

**Figure 6:**
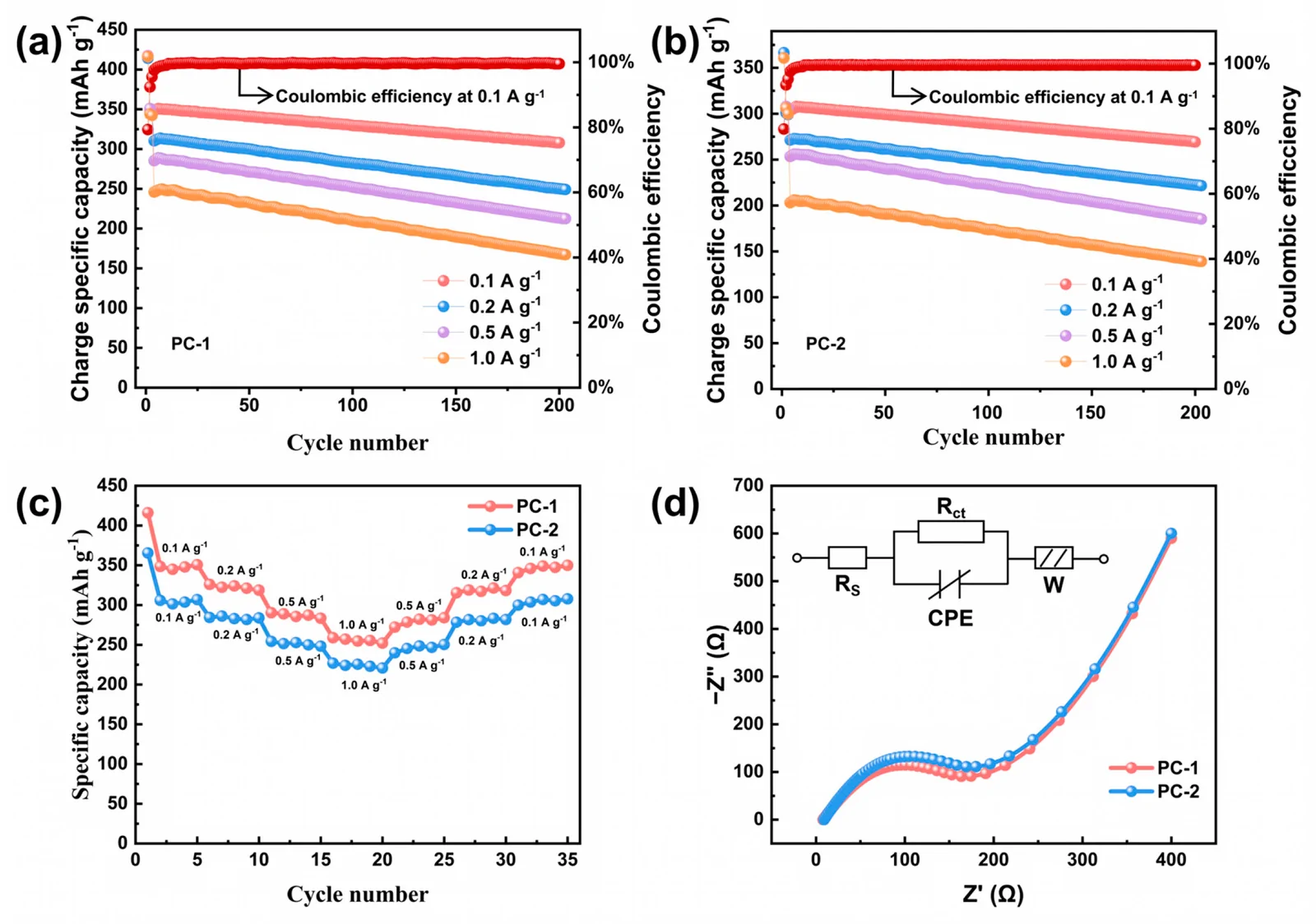
Cycling performance, rate capability, and electrochemical impedance spectroscopy (EIS) analysis of PC-1 and PC-2. (**a**,**b**) Cycling performance of PC-1 and PC-2, respectively, at 0.1, 0.2, 0.5, and 1 A g^−1^; (**c**) rate capability of PC-1 and PC-2 at different current densities; and (**d**) Nyquist plots of PC-1 and PC-2.

To evaluate the cycling stability of PC-1 and PC-2 at different current densities, their charge capacities were monitored throughout cycling. The initial discharge capacity contains irreversible contributions associated with SEI formation, electrolyte decomposition, and other interfacial side reactions. Therefore, the charge capacity was used in this study to evaluate the evolution of reversible capacity during long-term cycling. As shown in Figure 6a,b, PC-1 and PC-2 were each cycled for 200 cycles at 0.1, 0.2, 0.5, and 1 A g^−1^. For the tests at 0.2, 0.5, and 1 A g^−1^, the cells were initially activated for three cycles at 0.1 A g^−1^ to promote electrolyte wetting, initial SEI formation, and stabilization of the electrode interface. The current density was subsequently increased to the target value, and the first charge capacity after switching was used as the reference for calculating capacity retention. For cycling at 0.1 A g^−1^, the second-cycle charge capacity was used as the reference. The Coulombic efficiencies of both samples during cycling at 0.1 A g^−1^ are also presented in the upper part of the figure to evaluate their reaction reversibility during long-term cycling. For PC-1, the reference charge capacities at 0.1, 0.2, 0.5, and 1 A g^−1^ were 347.32, 310.28, 285.38, and 245.96 mAh g^−1^, respectively. After 200 cycles, the corresponding capacities were 307.86, 249.22, 212.52, and 167.21 mAh g^−1^, resulting in capacity retentions of 88.64%, 80.32%, 74.47%, and 67.98%, respectively. For PC-2, the charge capacities decreased from 304.69, 271.27, 253.41, and 217.33 mAh g^−1^ to 269.19, 221.60, 185.34, and 148.85 mAh g^−1^, respectively, corresponding to capacity retentions of 88.35%, 81.69%, 73.14%, and 68.49%. Based on three independently assembled cells, the reversible capacities after 200 cycles at 0.1 A g^−1^ were 307.8 ± 3.5 and 269.2 ± 2.7 mAh g^−1^ for PC-1 and PC-2, respectively (*n* = 3), confirming the reproducibility of their long-term cycling performance. The two samples exhibit similar overall capacity-retention values, indicating comparable long-term cycling stability. The capacity retention gradually decreases with increasing current density, which may be associated with enhanced electrode polarization and more pronounced Li^+^ transport limitations at higher current densities [58]. PC-1 maintains a higher reversible capacity than PC-2 at every investigated current density. This capacity difference occurs alongside multiple physicochemical differences between the selected samples, while their similar capacity-retention trends indicate comparable long-term cycling stability. Both samples exhibit a slight capacity increase during the initial stage of cycling, followed by a gradual decline. The initial increase may arise from progressive electrolyte wetting and activation of previously inaccessible lithium-storage sites, whereas the subsequent decay may be associated with interfacial evolution and the accumulation of electrode polarization [59,60].

Following the cycling-stability evaluation, rate-capability tests were conducted to further investigate the lithium-storage performance of PC-1 and PC-2 at different current densities. As shown in Figure 6c, the cells were sequentially cycled at 0.1, 0.2, 0.5, 1.0, 0.5, 0.2, and 0.1 A g^−1^ for five cycles at each current density, and the charge capacities were recorded. The average charge capacities of PC-1 at these successive stages were 361.64, 322.48, 287.11, 255.61, 279.61, 318.21, and 346.60 mAh g^−1^, respectively. The corresponding values for PC-2 were 316.64, 283.81, 251.37, 224.06, 246.21, 281.03, and 304.73 mAh g^−1^, respectively. As the current density increased from 0.1 to 1.0 A g^−1^, the capacities of both samples decreased stepwise, mainly because of increased electrode polarization and the reduced time available for Li^+^ transport and interfacial reactions at higher current densities [61]. PC-1 maintained a higher charge capacity at every investigated current density. This trend was observed together with its larger pore volume, more open mesoporous structure, and other differences in carbon-framework characteristics and ion-transport behavior [10,62]. When the current density was returned to 0.1 A g^−1^, the average capacities of PC-1 and PC-2 recovered to 95.84% and 96.24% of their respective initial-stage average values, demonstrating favorable rate reversibility and capacity-recovery capability for both samples [63]. The two samples exhibit similar rate-response trends, whereas PC-1 maintains a higher reversible capacity throughout the test.

Electrochemical impedance spectroscopy (EIS) was employed to further investigate the kinetic origin of the electrochemical-performance differences between PC-1 and PC-2, with particular emphasis on their interfacial reactions and Li^+^ transport behavior. As shown in Figure 6d, the Nyquist plots of both samples consist of a depressed semicircle in the medium- to high-frequency region followed by an inclined line in the low-frequency region. The spectra were fitted using the equivalent circuit R_s_–(R_ct_‖CPE)–W, and the corresponding fitted parameters are summarized in Table 3. The fitted R_s_ and R_ct_ values of PC-1 are approximately 8 and 150 Ω, respectively, whereas those of PC-2 are approximately 9 and 155 Ω, respectively. The similar R_S_ values suggest that the two cells have comparable electrolyte resistance, current-collector contact, and overall ohmic losses. In comparison, PC-1 exhibits a slightly lower R_ct_, consistent with the smaller medium- to high-frequency semicircle in its Nyquist plot. This result suggests that the PC-1 electrode encounters slightly lower kinetic resistance during charge transfer across the electrode/electrolyte interface. The Warburg coefficients (σW) of PC-1 and PC-2 were 79.12 and 81.01 Ω s^−1/2^, respectively. Their relatively small difference indicates that the two electrodes exhibit similar Li^+^ diffusion impedances. The slightly lower σW of PC-1 suggests reduced diffusion resistance in the low-frequency region and a modest advantage in Li^+^ transport within the electrode, consistent with the slightly higher Li^+^ diffusion coefficients of PC-1 over most of the potential range in the subsequent GITT measurements. The relatively low R_ct_ values of both electrodes may be associated with their interconnected three-dimensional carbon frameworks and relatively uniform interfacial reaction distributions. The continuous conductive frameworks can reduce interruptions in interparticle electron transport and contact losses, while improving local current distribution within the electrodes and mitigating interfacial charge accumulation and localized polarization, thereby facilitating charge transfer across the electrode/electrolyte interface [64]. Although the differences in the impedance parameters of the two samples were relatively limited, the lower R_ct_ and σW values of PC-1 indicate modestly improved interfacial charge-transfer and Li^+^ diffusion kinetics. These differences provide supporting evidence for the slightly improved electrochemical kinetics of PC-1 and contribute to the interpretation of its favorable lithium-storage performance.

**Figure 7:**
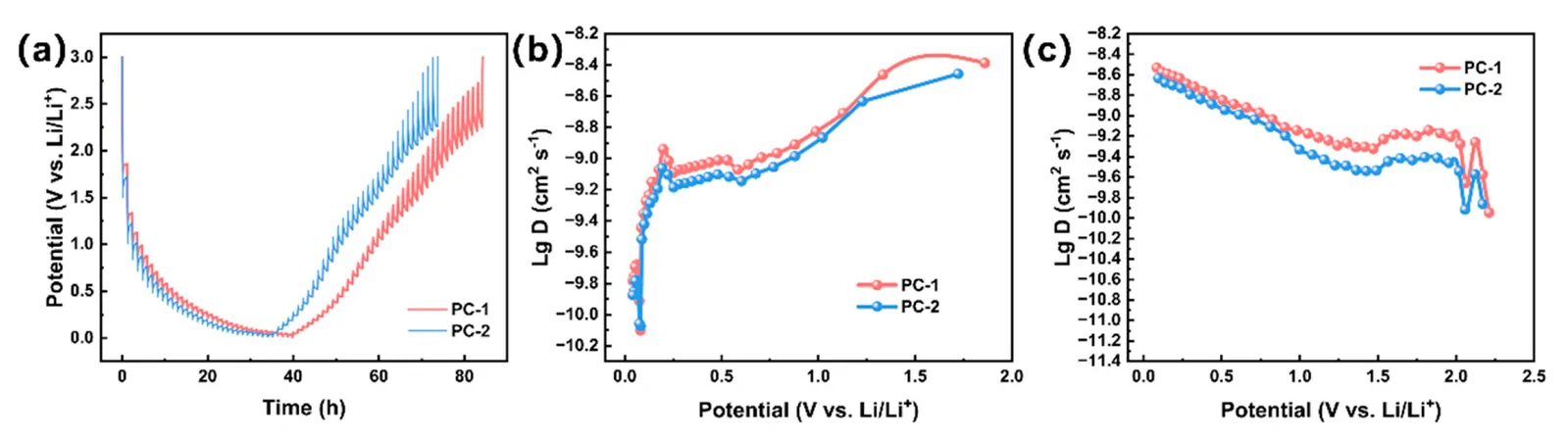
GITT results of the PC-1 and PC-2 electrodes. (**a**) Voltage-response profiles during lithiation and delithiation; (**b**) variation in the logarithmic Li^+^ diffusion coefficient (Lg D) with potential during lithiation; and (**c**) variation in Lg D with potential during delithiation.

To further evaluate the Li^+^ transport kinetics of PC-1 and PC-2 during lithiation and delithiation, the two electrodes were investigated using the galvanostatic intermittent titration technique (GITT). As shown in Figure 7a, both electrodes exhibit the characteristic response of repeated galvanostatic current pulses followed by open-circuit relaxation. The Li^+^ diffusion coefficient D was calculated according to Fick’s second law using Equation (3):

(3) $$D_{{Li}^{+}}=\frac{4}{\pi \tau }{\left(\frac{m_{B}}{\rho S}\right)}^{2}{\left(\frac{{△E}_{s}}{{△E}_{t}}\right)}^{2}$$

The variation in the Li^+^ diffusion coefficients of PC-1 and PC-2 with potential is shown in Figure 7b,c. The Lg D values of both electrodes exhibited pronounced fluctuations with potential, indicating that Li^+^ diffusion kinetics were closely related to the lithium-storage state within the electrodes. During lithiation, the Li^+^ diffusion coefficients of PC-1 and PC-2 ranged from 7.91 × 10^−11^ to 4.10 × 10^−9^ cm^2^ s^−1^ and from 8.42 × 10^−11^ to 3.49 × 10^−9^ cm^2^ s^−1^, respectively. During delithiation, the corresponding values ranged from 1.13 × 10^−10^ to 2.93 × 10^−9^ cm^2^ s^−1^ and from 1.21 × 10^−10^ to 2.33 × 10^−9^ cm^2^ s^−1^, respectively. The potential-dependent fluctuations in the diffusion coefficients can be attributed to changes in the dominant lithium-storage sites and local diffusion resistance as Li^+^ is progressively accommodated at or released from defects, edges, pore-wall sites, and locally short-range-ordered carbon layers with different accessibility and binding strengths. Changes in site occupancy and local concentration gradients therefore result in a nonuniform apparent diffusion response during lithiation and delithiation [55]. Throughout the lithiation/delithiation process, PC-1 exhibited slightly higher Li^+^ diffusion coefficients than PC-2 over most of the potential range, indicating more favorable ion-transport kinetics. The potential-dependent variation in D further supports this interpretation. Although both electrodes display similar diffusion-coefficient profiles, suggesting a comparable lithium-storage mechanism, PC-1 maintains a modest diffusion advantage across most lithiation and delithiation states. This trend is consistent with its slightly lower σW, jointly indicating reduced Li^+^ transport resistance throughout the electrochemical process rather than only at a specific potential. In contrast, the relatively small difference in R_ct_ alone is insufficient to explain the better electrochemical performance of PC-1 and only indicates a slight advantage in interfacial charge-transfer kinetics. Therefore, the higher reversible capacity and greater capacity output of PC-1 at elevated current densities are correlated with the combined physicochemical differences between PC-1 and PC-2, including pore architecture, specific surface area, carbon-layer arrangement, and local structural ordering, together with the modest differences observed in charge-transfer and Li^+^ diffusion kinetics.

The electrochemical results show that PC-1 and PC-2 exhibit similar lithium-storage behavior, comparable initial Coulombic efficiencies, cycling-capacity retention, and capacity recovery during rate testing. These similarities are consistent with the XRD and Raman results, which demonstrate that both samples are predominantly amorphous, defect-rich carbon materials with low degrees of graphitization. Their comparable carbon-framework characteristics therefore account for the similar electrochemical reaction profiles, reversibility, and long-term cycling trends. Nevertheless, the differences in their physicochemical structures lead to distinguishable capacity outputs and kinetic behavior. The larger d_002_ value of PC-1 (0.3842 nm) compared with that of PC-2 (0.3736 nm) indicates a less compact carbon-layer arrangement, which may facilitate Li^+^ insertion and extraction within the locally short-range-ordered carbon domains. Meanwhile, the lower ID/IG ratio of PC-1 (1.4080) relative to PC-2 (1.5256) suggests a slightly higher degree of local structural ordering, which may contribute to more continuous electron transport and is consistent with its slightly lower charge-transfer resistance. Although the higher defect density of PC-2 may provide additional potential lithium-storage sites, the electrochemical results indicate that defect density alone does not determine the effectively accessible capacity. Differences in pore structure may be associated with variations in site accessibility and ion transport, together with other synthesis-derived structural characteristics. PC-1 possesses a higher BET specific surface area, a substantially larger total pore volume, a larger average mesopore diameter, and a more open and interconnected mesoporous framework. These characteristics may favor electrolyte infiltration, site accessibility, and Li^+^ transport and are consistent with the electrochemical behavior observed for PC-1. The larger interfacial area of PC-1 may also promote slightly more extensive initial interfacial reactions, which is consistent with its marginally lower initial Coulombic efficiency; however, the limited difference between the two samples indicates that both form comparably stable electrode interfaces. Therefore, the electrochemical-performance difference between PC-1 and PC-2 is associated with combined variations in carbon-layer arrangement, local structural ordering, pore accessibility, and other synthesis-dependent characteristics. These results establish a direct correspondence between the characteristics of the selected candidates and their observed lithium-storage performance and demonstrate the application relevance of the DOE-assisted candidate-selection strategy.

A literature benchmark was conducted by comparing the initial reversible specific capacity, ICE, and cycling performance of PC-1 with those of recently reported RF-derived porous carbon anodes, as summarized in Table 4. As shown in Table 4, PC-1 exhibits a relatively high initial reversible specific capacity among the recently reported RF-derived porous carbon anodes included in the comparison. Notably, its initial reversible capacity of 416.67 mAh g^−1^ moderately exceeds the theoretical capacity of graphite (372 mAh g^−1^), which is based on the formation of LiC_6_ through conventional intercalation. The additional reversible capacity may be attributed to lithium storage at defect and edge sites, interfacial storage on accessible pore surfaces, and lithium insertion into the expanded and disordered carbon layers of PC-1. Its predominantly sloping voltage profile is also qualitatively similar to that commonly observed for hard-carbon anodes, indicating a distribution of lithium-storage environments rather than a single graphite-like intercalation process. Its ICE is also higher than those of most of the listed materials, indicating relatively limited irreversible lithium consumption during the initial cycle. Together with its cycling performance, these results demonstrate that PC-1 achieves a favorable balance among initial reversible capacity, initial Coulombic efficiency, and long-term cycling stability. It should be noted that the current densities, cycle numbers, electrode conditions, and testing protocols vary among the different studies. Therefore, this comparison is intended to provide a general performance benchmark rather than a strict ranking of the reported materials. The DOE-selected PC-1 delivers competitive and well-balanced lithium-storage performance without relying on additional heteroatom doping, metal incorporation, or complex composite construction. From a practical perspective, the synthesis is based on conventional RF sol–gel processing, drying, and carbonization, while the DOE approach provides a structured basis for factor screening and candidate selection and can thereby reduce reliance on empirical trial-and-error. However, the use of freeze drying, prolonged gelation and aging, and high-temperature carbonization may increase processing time, energy consumption, and cost. In addition, the present study is limited to laboratory-scale preparation and lithium-metal half-cell testing; batch consistency, high-mass-loading electrodes, volumetric performance, and full-cell behavior require further evaluation.

## 3. Conclusions

In this study, a mixed-level Design of Experiments (DOE) was employed to systematically investigate the effects of nominal solid content, gelation temperature, R/C ratio, combined gelation and acid-washing/aging times, and drying method on the pore structure of RF-derived porous carbons. The ANOVA results identified the R/C ratio and gelation temperature as the principal factors affecting the BET specific surface area and total pore volume, whereas the combined gelation and acid-washing/aging times exhibited the most significant main effect on the dominant pore size within the present dataset. Based on the multi-response evaluation of pore-structure characteristics and processing practicality, Runs 24 and 7 were selected as representative candidate conditions for the preparation of PC-1 and PC-2, respectively. Subsequent structural and electrochemical analyses showed that PC-1 possessed a more accessible pore structure and exhibited a higher reversible capacity and more favorable ion-transport behavior than PC-2. These results indicate an association between the overall physicochemical characteristics of the selected candidates and their lithium-storage performance while supporting the usefulness of DOE-assisted screening for identifying influential preparation factors and representative candidate conditions.

Overall, this study establishes an integrated DOE-assisted screening framework linking preparation parameters, pore-structure responses, representative-candidate selection, and lithium-storage performance evaluation for RF-derived porous carbon materials. Given the relatively limited predicted R^2^ values and the significant lack-of-fit of the present main-effects models, these models are interpreted primarily as exploratory tools for main-effect screening and relative factor ranking rather than as quantitatively validated models for predicting globally optimal processing conditions. Accordingly, the present results support application-oriented candidate selection within the experimentally investigated design space but should not be interpreted as demonstrating precise predictive optimization or direct scale-up capability. Future studies incorporating additional replicate experiments, interaction terms, expanded design spaces, and independent validation conditions will be valuable for developing more robust predictive models and further evaluating process optimization and scale-up potential.

## 4. Materials and Methods

### 4.1. Preparation of RF-Derived Porous Carbons

RF-derived porous carbons were prepared using resorcinol and a 37 wt.% formaldehyde solution as the precursors and sodium carbonate (Na_2_CO_3_) as the basic catalyst. Resorcinol and formaldehyde were mixed at a molar ratio of 1:2, and the nominal solid content was adjusted by varying the amount of deionized water. The R/C ratio was defined as the molar ratio of resorcinol (R) to Na_2_CO_3_ catalyst (C), i.e., R/C = n(resorcinol)/n(Na_2_CO_3_). The mixture was stirred at room temperature to obtain a homogeneous RF precursor solution. Under the alkaline catalytic conditions provided by Na_2_CO_3_, resorcinol first reacted with formaldehyde through hydroxymethylation, followed by condensation reactions between the hydroxymethylated species to form an increasingly cross-linked three-dimensional RF polymer network connected mainly by methylene and methylene-ether bridges. Subsequently, the precursor solution was sealed and allowed to undergo gelation at the designated temperature. The resulting RF wet gels were acid-washed and aged in a 5% acetic acid solution. After solvent exchange, the gels were dried using the designated drying methods to obtain dried RF organic gels. Finally, the samples were carbonized at 1050 °C under an inert atmosphere to obtain RF-derived porous carbons. Previous studies have shown that RF-derived carbons can be successfully obtained over a broad carbonization range of approximately 700–1100 °C, with increasing temperature generally favoring carbonization and electronic conductivity. Temperatures around 1000–1050 °C have also been widely employed for RF-derived porous carbons and provide an appropriate balance between sufficient carbonization, electrical conductivity, and preservation of the porous framework [70,71,72]. Carbonization at approximately 1050 °C has been directly adopted in previous studies for the preparation of RF-derived carbon materials [73]. Accordingly, 1050 °C was adopted as a fixed carbonization condition for all samples to ensure consistency and comparability within the DOE.

### 4.2. Design of Experiments and Statistical Analysis

To systematically evaluate the effects of the preparation parameters on the pore structure of RF-derived porous carbons, a mixed-level experimental design was employed. Five experimental factors were investigated: nominal solid content, gelation temperature, the molar ratio of resorcinol to catalyst (R/C), the combined gelation and acid-washing/aging times, and drying method. Here, R/C denotes the molar ratio of resorcinol to Na_2_CO_3_. The nominal solid content was controlled by varying the volume of deionized water added to the precursor solution. Deionized water volumes of 9, 20, and 40 mL corresponded to nominal solid contents of 34.3, 22.1, and 13.4 wt.%, respectively. The gelation temperatures were set at 40, 60, and 80 °C, whereas the R/C ratios were set at 100, 200, 400, and 1000. The combined gelation and acid-washing/aging times were set at (8 + 4), (16 + 8), (24 + 12), and (48 + 24) h, where the first and second values represent the gelation and acid-washing/aging durations, respectively. The drying methods included ambient-pressure drying and freeze drying. The investigated factors and their corresponding levels are summarized in Table 5. Additionally, the synthesis parameters, processing conditions, and corresponding pore-structure characteristics of all experimental runs are comprehensively summarized in Table S8.

The experimental matrix was generated using the DOE module in JMP 19 statistical software (JMP Statistical Discovery LLC, Cary, NC, USA), with a main-effects-only model containing all five factors specified as the target model. The experimental matrix was analyzed using a main-effects model containing all five investigated factors to estimate their average effects and relative importance within the experimental design space. Because the adopted analysis was centered on the estimation of main effects, interaction terms were not included in the primary statistical model, and the resulting factor contributions should therefore be interpreted as main-effect screening results rather than as a complete description of the relationships among the processing variables. The adopted experimental design and available valid runs did not provide sufficient independent information for reliable estimation of the major two-factor interaction effects. Therefore, interaction terms were not interpreted quantitatively, and the DOE results were restricted to exploratory main-effect screening and relative factor ranking within the investigated design space. The design comprised 32 main experimental runs and four additional replicate runs, which were included to estimate the experimental error and preliminarily assess experimental repeatability. Gel formation was additionally analyzed as a binary process-feasibility response, with successful gelation coded as 1 and gelation failure coded as 0. The numbers and proportions of successful and failed runs were summarized for each factor level. Fisher’s exact test was used for the two-level drying-method factor, whereas the Fisher–Freeman–Halton exact test was used for factors containing more than two levels. Because only five failure events were observed across the five mixed-level factors and several factor levels contained no failure events, a multivariable binary logistic model would be susceptible to unstable coefficient estimates and separation. Therefore, the binary response was evaluated using descriptive success rates and exploratory factor-wise exact tests rather than a multivariable regression model. Continuous pore-structure responses were analyzed only for successfully gelled samples, and no statistical imputation was performed for the failed runs because the corresponding pore-structure responses were undefined. All experimental combinations were randomly selected from the complete DOE matrix and performed according to a randomized run sequence. The samples assigned to ambient-pressure drying and freeze drying were therefore not prepared as two sequential, non-randomized batches. Instead, each sample was subjected to the drying method specified by its corresponding DOE condition. This randomized arrangement distributed the different factor levels throughout the experimental sequence, thereby minimizing the possibility that potential batch, run-order, or time-dependent variations were systematically associated with a particular drying method. The Run IDs and arrangement of preparation parameters presented in Table S8 were organized solely for clear presentation and data traceability and do not represent the actual chronological sequence of the experiments. In addition, drying method was included in the DOE analysis as a categorical factor to account for the systematic differences associated with the two drying procedures. Therefore, the combined use of randomized experimental execution and explicit inclusion of drying method in the statistical model provided a structured basis for evaluating the drying-method factor while minimizing potential batch-related influences.

The BET specific surface area, total pore volume, and dominant pore diameter derived from the desorption branch were selected as the principal pore-structure responses. The main effects and relative contributions of the preparation factors were analyzed to identify and rank the dominant factors, while the pore structures obtained under different combinations of factor levels were also compared. Statistical significance and practical significance were evaluated separately when interpreting and ranking the investigated factors. Statistical significance was assessed using the ANOVA *p*-values, with *p* < 0.05 considered statistically significant and 0.05 ≤ *p* < 0.10 considered marginally significant. The contribution rate derived from the sums of squares was used to describe the relative proportion of model-attributed variation associated with each main effect within the adopted model and was not treated as an independent test of statistical significance. Practical significance was evaluated by jointly considering the magnitude and direction of the observed factor-level changes, the distributions and measured responses of the individual experimental runs, gelation feasibility, and processing practicality. Given the significant lack-of-fit and limited predictive performance of the models, these criteria were used for exploratory screening and relative factor ranking within the investigated design space rather than for quantitative prediction of globally optimal conditions.

For the multi-response evaluation, the desirability functions were defined using the Response Limits settings in JMP 19. Each pore-structure response was treated as a target-type response, and its measured value was transformed into an individual desirability ranging from 0 to 1. In JMP, the individual desirability function for a target-type response is represented by a smooth piecewise function defined by the corresponding low, middle, and high control points, with the highest desirability assigned to the target region and progressively lower desirability assigned as the response deviates from the desired value or range. The BET specific surface area, total pore volume, and dominant pore size were assigned equal importance (1:1:1), so that no single pore-structure response was preferentially weighted in the overall evaluation. The individual response desirabilities were evaluated according to the predefined screening criteria, and the overall desirability was used as the statistical basis for identifying the primary candidate. Candidate selection additionally incorporated processing practicality as an engineering consideration. Specifically, Run 24 was selected as the primary candidate based principally on the multi-response desirability evaluation, whereas Run 7 was selected as an alternative candidate by additionally considering the practical advantage of ambient-pressure drying. The selected candidates were subsequently subjected to structural characterization and electrochemical performance evaluation.

### 4.3. Material Characterization

The morphology and microstructure of the RF-derived porous carbons were characterized by scanning electron microscopy (SEM; Regulus 8100, Hitachi, Japan) and transmission electron microscopy (TEM; Talos F200X G2, FEI, USA). Their crystallographic structures were analyzed by X-ray diffraction (XRD; SmartLab SE, Rigaku, Japan). Raman spectra were recorded using a Raman spectrometer (LabRAM Odyssey, HORIBA, Japan) to evaluate the structural defects and degree of graphitization of the derived porous carbons. The pore structures were analyzed by N_2_ adsorption–desorption measurements at 77 K using a Micromeritics ASAP 2460 analyzer (Micromeritics, USA). Before measurement, the samples were degassed under vacuum at 250 °C for 6 h. The specific surface area was calculated using the Brunauer–Emmett–Teller (BET) method over P/P_0_ = 0.05–0.30, while the total pore volume was determined at P/P_0_ ≈ 0.99. The pore-size distributions and average pore diameters were calculated from the desorption branch using the Barrett–Joyner–Halenda (BJH) method. XPS measurements were performed using a Thermo Scientific K-Alpha spectrometer (Thermo Fisher Scientific, USA) with a monochromatic Al Kα X-ray source. The binding energies were calibrated using the C–C/C=C peak at 284.8 eV, and the high-resolution spectra were fitted using a Shirley background and mixed Gaussian–Lorentzian line shapes. The peak positions were constrained according to the corresponding chemical assignments, and comparable peak-width criteria were applied to PC-1 and PC-2. TG–DSC measurements were conducted using a Hitachi STA200 simultaneous thermal analyzer (Hitachi, Japan). Approximately 10 mg of each sample was heated from room temperature to 1000 °C at 10 °C min^−1^ under flowing air at 100 mL min^−1^. For SEM and TEM characterization, multiple randomly selected regions of each sample were examined to confirm that the observed morphological and microstructural features were representative. XRD, Raman, N_2_ adsorption–desorption, XPS, and TG–DSC measurements were performed on representative samples obtained from the corresponding synthesis conditions. For the reproducibility evaluation of the selected preparation conditions, PC-1 and PC-2 were independently re-prepared three times, and their BET specific surface area, total pore volume, and dominant pore size are reported as the mean ± standard deviation (*n* = 3). Unless otherwise stated, these physicochemical characterization results are presented as representative measurements rather than statistical replicates.

### 4.4. Electrochemical Measurements

RF-derived porous carbon, acetylene black, and poly(vinylidene fluoride) (PVDF) were mixed at a mass ratio of 8:1:1, using N-methyl-2-pyrrolidone (NMP) as the solvent to prepare a homogeneous electrode slurry. The slurry was coated onto a copper-foil current collector with a wet-film thickness of approximately 50 μm and subsequently dried to obtain the working electrodes. The active-material mass was approximately 1.6 mg per electrode. Coin-type half-cells were subsequently assembled in an argon-filled glovebox using the RF-derived porous carbon electrode as the working electrode and lithium-metal foil as the counter/reference electrode. Galvanostatic charge–discharge and galvanostatic intermittent titration technique (GITT) measurements were performed using a LAND battery-testing system (LAND, China). Cyclic voltammetry and electrochemical impedance spectroscopy measurements were conducted using an electrochemical workstation (CHI660, Chenhua, China). All electrochemical measurements were independently repeated using at least three cells assembled under identical conditions to verify reproducibility. Unless otherwise stated, representative curves corresponding closely to the average behavior of the independent cells are presented, while the reproducibility of key electrochemical parameters is reported as the mean ± standard deviation (*n* = 3).

## Figures and Tables

**Table 1:** Multi-response evaluation tailored to the requirements of lithium-ion battery anodes.

Run ID	Drying Method	Deionized Water Volume (mL)	Temperature (°C)	R/C	Combined Gelation and Acid-Washing/Aging Times (h)	BET Specific Surface Area (m^2^ g^−1^)	Total Pore Volume (cm^3^ g^−1^)	Dominant Pore Size (nm)	Overall Desirability Score
24	Freeze drying	9	80	400	48 + 24	737.5949	1.4750	9.2353	0.8558
27	Freeze drying	20	80	400	16 + 8	658.6353	0.9879	7.5651	0.7687
7	Ambient-pressure drying	9	60	1000	24 + 12	640.5655	0.4818	6.1128	0.5984
16	Ambient-pressure drying	40	80	400	24 + 12	367.9637	0.5841	8.1458	0.5262
26	Freeze drying	40	60	400	24 + 12	461.0496	0.3555	3.8047	0.4673

**Note:** Equal importance was assigned to the BET specific surface area, total pore volume, and dominant pore size in the multi-response evaluation. Run 24 was selected as the primary candidate principally on the basis of the overall desirability evaluation, whereas Run 7 was selected as a secondary candidate by additionally considering the practical advantage of ambient-pressure drying. Therefore, the selection of Run 7 was not based solely on its overall desirability ranking.

**Table 2:** Preparation conditions of the selected RF-derived porous carbon samples.

Corresponding Run	Sample Designation	Sample Role	Nominal Solid Content (wt.%)	Gelation Temperature (°C)	R/C	Gelation + Acid-Washing/Aging Times (h)	Drying Method
Run 24	PC-1	Primary preparation route	34.3	80	400	48 + 24	Freeze drying
Run 7	PC-2	Alternative preparation route	34.3	60	1000	24 + 12	Ambient-pressure drying

**Table 3:** Fitted impedance parameters of PC-1 and PC-2.

Sample	R_s_ (Ω)	R_ct_ (Ω)	σW (Ω·s^−1/2^)
PC-1	8	150	79.12
PC-2	9	155	81.01

**Table 4:** Comparison of the electrochemical performance of PC-1 with selected representative carbon-based anode materials for lithium-ion batteries.

Ref.	Initial Reversible Specific Capacity (mAh g^−1^)	ICE	Cycling Performance
[16]	210.42	70.14%	77.5% after 100 cycles at 0.1 C
[65]	288	63.15%	96.9% after 100 cycles at 0.1 C
[66]	417.8	74.8%	74.3% after 200 cycles at 0.5 A g^−1^
[67]	386	50.5%	97.1% after 20 cycles at 0.1 C
[68]	579	38.5%	86.7% after 100 cycles at 0.5 A g^−1^
[69]	454.6	83.14%	96.91% after 200 cycles at 1.0 A g^−1^
This article	416.67	79.31%	88.64% after 200 cycles at 0.1 A g^−1^

**Table 5:** Experimental factors and levels used in the mixed-level DOE for the preparation of RF-derived porous carbons.

Factor	Parameter	Level 1	Level 2	Level 3	Level 4
A	Nominal solid content (wt.%)	34.3	22.1	13.4	—
B	Temperature (°C)	40	60	80	—
C	R/C	100	200	400	1000
D	Gelation + acid-washing/aging times (h)	8 + 4	16 + 8	24 + 12	48 + 24
E	Drying method	Ambient-pressure drying	Freeze drying	—	—

## Data Availability

All data generated or analyzed during this study are included in this article and its Supplementary Materials.

## Supplementary Materials

The following supporting information can be downloaded at https://www.mdpi.com/article/10.3390/gels12090801/s1, Figure S1: NLDFT pore-size distributions of PC-1 and PC-2. Table S1. Gel-formation feasibility analysis of the mixed-level DOE: (a) Experimental runs that failed to form gels; and (b) Factor-level summary of gelation outcomes and exploratory exact-test results; Table S2. Replicate-point statistics and experimental error; Table S3. Model adequacy and residual diagnostics for the pore-structure response; Table S4. ANOVA results for the significance of the investigated factors; Table S5. Level-mean analysis of the BET specific surface area; Table S6. Level-mean analysis of the total pore volume; Table S7. Level-mean analysis of the dominant pore size; Table S8. Synthesis parameters, processing conditions, and pore-structure characteristics of all synthesized RF-derived porous carbon samples.
