# Supplementary material for: Pore Structure Regulation of RF-Derived Porous Carbons Using a Mixed-Level Design of Experiments for Lithium-Ion Battery Anode Materials
Source: Gels. 2026 Sep 2;12(9):801. doi: 10.3390/gels12090801 (PMC13606249; doi:10.3390/gels12090801)
Supplement: Supplementary file 1 [file gels-12-00801-s001.zip › gels-4534113-supplementary.pdf]

Supplementary Materials

Table S1. Gel-formation feasibility analysis of the mixed-level DOE: (a) experimental runs that failed to form gels and (b) factor-level summary of gelation outcomes and exploratory exact-test results.

Table S1 (a). Experimental runs that failed to form gels.

Run ID	Drying Method	Deionized Water Volume (mL)	Temperature (°C)	R/C	Combined Gelation and Acid-Washing/Aging Times (h)
1	Ambient-pressure drying	40	40	400	16+8
6	Ambient-pressure drying	40	60	1000	8+4
17	Freeze drying	20	40	400	8+4
21	Freeze drying	40	40	1000	24+12
23	Freeze drying	40	80	1000	48+24

Table S1 (b). Factor-level summary of gelation outcomes and exploratory exact-test results.

Factor	Level	Total Runs	Successful Runs	Failed Runs	Gelation Success Rate (%)	Exploratory Exact p-Value
Drying method	Ambient-pressure drying	18	16	2	88.9	1.000
	Freeze drying	18	15	3	83.3	
Deionized water volume (mL)	9	15	15	0	100.0	0.0080
	20	11	10	1	90.9	
	40	10	6	4	60.0	
Temperature (°C)	40	11	8	3	72.7	0.4788
	60	11	10	1	90.9	
	80	14	13	1	92.9	
R/C	100	9	9	0	100.0	0.0499
	200	10	10	0	100.0	
	400	9	7	2	77.8	
	1000	8	5	3	62.5	
Combined gelation and acid-washing/aging times (h)	8+4	8	6	2	75.0	0.8448
	16+8	7	6	1	85.7	
	24+12	10	9	1	90.0	
	48+24	11	10	1	90.9	

Note: Gelation success or failure was treated as a binary process-feasibility response. The p-value for drying method was calculated using Fisher's exact test, whereas the p-values for the multilevel factors were calculated using the Fisher–Freeman–Halton exact test. These factor-wise tests were exploratory and were not used to infer independent causal effects. In particular, all three gelation failures observed at R/C = 1000 occurred simultaneously at a deionized water volume of 40 mL, indicating that the apparent R/C-related pattern cannot be separated from the low-solid-content condition using the available failure events.

Table S2. Replicate-point statistics and experimental errors.

Drying Method	Response	Mean	Standard Deviation	Coefficient of Variation (%)	Range
Freeze drying	BET specific surface area (m^2 g^{-1})	460.8906	11.1127	2.4111	15.7157
Freeze drying	Total pore volume (cm^3 g^{-1})	0.3475	0.0217	6.2366	0.0307
Freeze drying	Dominant pore size (nm)	3.4985	0.0739	2.1122	0.1045
Ambient-pressure	BET specific	474.9784	19.2775	4.0586	27.2625

Drying Method	Response	Mean	Standard Deviation	Coefficient of Variation (%)	Range
drying	surface area (m^2 g^{-1})				
Ambient-pressure drying	Total pore volume (cm^3 g^{-1})	0.3786	0.0026	0.694	0.0037
Ambient-pressure drying	Dominant pore size (nm)	3.5046	0.0182	0.5185	0.0257

Table S3. Model adequacy and residual diagnostics for the pore-structure response

Response	R^2	Adjusted R^2	Predicted R^2	Shapiro–Wilk p	Breusch-Pagan p	Lack-of-fit F	Lack-of-fit p
In (BET)	0.701	0.528	0.202	0.236	0.265	640.00	0.0016
In (total pore volume)	0.746	0.599	0.302	0.978	0.271	418.59	0.0024
In (pore size)	0.612	0.388	-0.127	0.010	0.545	1035.26	0.0010

Table S4. ANOVA results for the significance of the investigated factors.

Response	Factor	Contribution Rate (%)	p-Value	Statistical Significance
BET specific surface area	R/C	39.5	0.0013	Significant
BET specific surface area	Temperature	25.2	0.004	Significant
BET specific surface area	Deionized water volume	2.2	0.5234	Not significant
BET specific surface area	Combined gelation and acid-washing/aging times	1.2	0.8742	Not significant
BET specific surface area	Drying method	0.1	0.8522	Not significant
Total pore volume	R/C	39.7	0.0006	Significant
Total pore volume	Temperature	28.6	0.0012	Significant
Total pore volume	Deionized water volume	1.2	0.6729	Not significant
Total pore volume	Combined gelation and acid-washing/aging times	1.9	0.7298	Not significant
Total pore volume	Drying method	1	0.413	Not significant
Dominant pore size	Combined gelation and acid-washing/aging times	24	0.0365	Significant
Dominant pore size	R/C	19	0.0709	Marginally significant
Dominant pore size	Deionized water volume	9.2	0.1616	Not significant
Dominant pore size	Temperature	4.1	0.4283	Not significant
Dominant pore size	Drying method	0	0.9751	Not significant

Note: Factors with $p < 0.05$ were considered statistically significant, whereas those with $0.05 \leq p < 0.10$ were considered marginally significant. The remaining contribution corresponds primarily to residual and experimental-error variation.

Table S5. Level-mean analysis of the BET specific surface area.

Factor	Level Associated with the Highest Mean Response	Mean BET Specific Surface Area (m^2 g^{-1})
Drying method	Freeze drying	334.1987
Deionized water volume (mL)	9	313.8387
Temperature (°C)	80	386.1644
R/C	1000	575.1336
Combined gelation and acid-washing/aging times (h)	16+8	395.2782

Table S6. Level-mean analysis of the total pore volume.

Factor	Level Associated with the Highest Mean Response	Mean Total Pore Volume (cm^3 g^{-1})
Drying method	Freeze drying	0.4204
Deionized water volume (mL)	9	0.3423
Temperature (°C)	80	0.4743
R/C	1000	0.6474
Combined gelation and acid-washing/aging times (h)	16+8	0.4117

Table S7. Level-mean analysis of the dominant pore size.

Factor	Level Associated with the Highest Mean Response	Mean Dominant Pore Size (nm)
Drying method	Ambient-pressure drying	6.8628
Deionized water volume (mL)	20	8.5493
Temperature (°C)	40	9.3105
R/C	1000	11.1631
Combined gelation and acid-washing/aging times (h)	24+12	10.8584

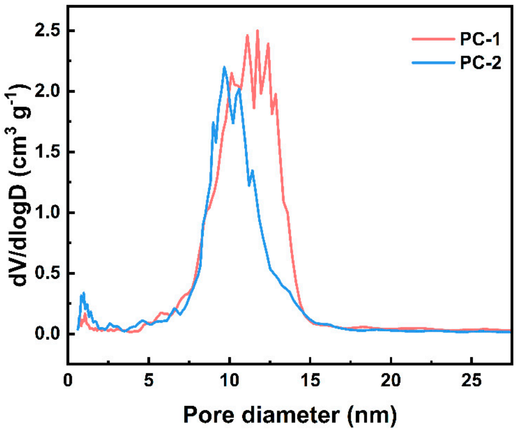

Figure S1. NLDFT pore-size distributions of PC-1 and PC-2.

Table S8. Synthesis parameters, processing conditions, and pore-structure characteristics of all synthesized RF-derived porous carbon samples.

Run ID	Drying Method	Water Volume (mL)	Temperature (℃)	R/C	Gelation/Acid-Aging Time (h)	Remarks	Dominant Pore Size (nm)	Specific Surface Area (m^2 g^{-1})	Total Pore Volume (cm^3/g)
1	Ambient-Pressure Drying	40	40	400	16+8	Gelation Failure			
2	Ambient-Pressure Drying	20	60	400	8+4		3.8670	175.2807	0.0882
3	Ambient-Pressure Drying	40	40	100	16+8		3.4888	199.7605	0.1034
4	Ambient-Pressure Drying	20	80	100	48+24		2.7755	96.4120	0.0563
5	Ambient-Pressure Drying	9	80	100	24+12		5.1245	176.4493	0.0903
6	Ambient-Pressure Drying	40	60	1000	8+4	Gelation Failure			
7	Ambient-Pressure Drying	9	60	1000	24+12		6.1128	640.5655	0.4818
8	Ambient-Pressure Drying	20	40	1000	48+24		9.1844	310.1996	0.1583
9	Ambient-Pressure Drying	9	40	1000	24+12		13.7959	274.9424	0.1668
10	Ambient-Pressure Drying	40	80	200	8+4		3.2349	198.8313	0.1080
11	Ambient-Pressure Drying	20	40	200	24+12		33.2523	115.3608	0.0591
12	Ambient-Pressure Drying	9	60	200	8+4		3.8031	86.2632	0.0421
13	Ambient-Pressure Drying	20	80	200	16+8		4.2417	117.7667	0.0672
14	Ambient-Pressure Drying	20	60	100	8+4		2.7670	88.3743	0.0420
15	Ambient-Pressure Drying	9	40	100	48+24		4.9557	4.1749	0.0049
16	Ambient-Pressure Drying	40	80	400	24+12		8.1458	367.9637	0.5841
17	Freeze Drying	20	40	400	8+4	Gelation Failure			
18	Freeze Drying	40	60	200	24+12		3.7668	140.5248	0.0637
19	Freeze Drying	9	80	1000	24+12		14.1187	682.2727	1.2842
20	Freeze Drying	40	80	100	16+8		3.3975	112.4736	0.0604

Run ID	Drying Method	Water Volume (mL)	Temperature (℃)	R/C	Gelation/Acid-Aging Time (h)	Remarks	Dominant Pore Size (nm)	Specific Surface Area (m^2 g^{-1})	Total Pore Volume (cm^3/g)
21	Freeze Drying	40	40	1000	24+12	Gelation Failure			
22	Freeze Drying	9	40	200	16+8		2.9295	70.0582	0.0357
23	Freeze Drying	40	80	1000	48+24	Gelation Failure			
24	Freeze Drying	9	80	400	48+24		9.2353	737.5949	1.4750
25	Freeze Drying	20	60	100	24+12		4.8588	51.9717	0.0631
26	Freeze Drying	40	60	400	24+12		3.8047	461.0496	0.3555
27	Freeze Drying	20	80	400	16+8		7.5651	658.6353	0.9879
28	Freeze Drying	9	60	400	8+4		3.2985	70.9670	0.0337
29	Freeze Drying	9	40	400	48+24		3.5238	43.1824	0.0417
30	Freeze Drying	20	40	100	8+4		3.3534	45.4093	0.0381
31	Freeze Drying	20	60	1000	16+8		14.0928	967.6877	1.1457
32	Freeze Drying	9	60	100	48+24		5.6179	49.3714	0.0263
33	Ambient-Pressure Drying	9	80	200	48+24	Replicate Run P1-1	3.4917	488.6097	0.3805
34	Ambient-Pressure Drying	9	80	200	48+24	Replicate Run P1-2	3.5174	461.3472	0.3768
35	Freeze Drying	9	80	200	48+24	Replicate Run P2-1	3.5507	453.0328	0.3629
36	Freeze Drying	9	80	200	48+24	Replicate Run P2-2	3.4462	468.7485	0.3322
